# Supplementary material for: Trans-eQTL mapping in gene sets identifies network effects of genetic variants
Source: Cell Genom. 2024 Apr 1;4(4):100538. doi: 10.1016/j.xgen.2024.100538 (PMC11019359; doi:10.1016/j.xgen.2024.100538)
Supplement: Document S3. Article plus supplemental information [file mmc19.pdf]

Cell Genomics

Article

Trans-eQTL mapping in gene sets identifies network effects of genetic variants

Graphical abstract

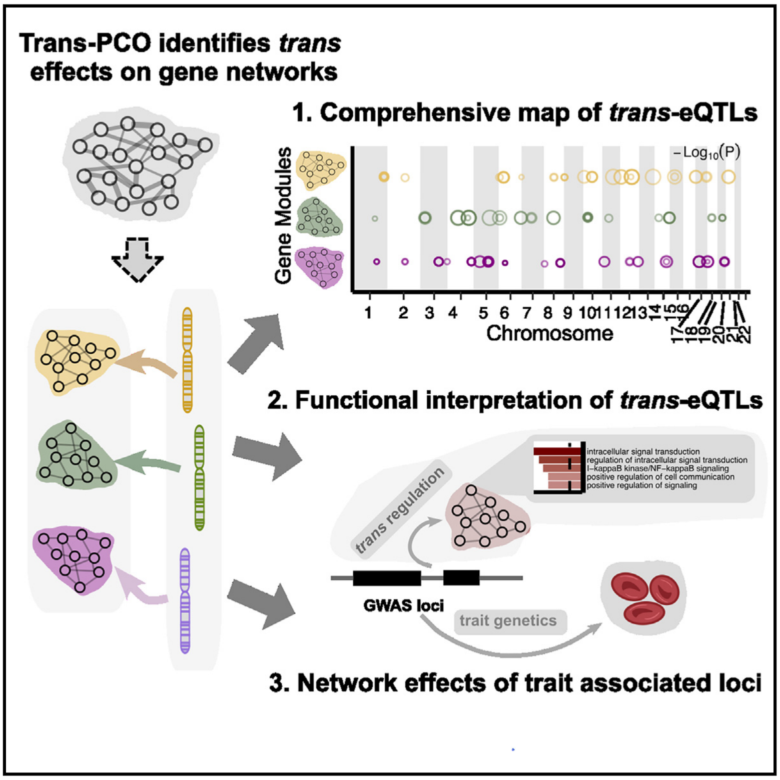

Highlights

- *Trans*-PCO outperforms existing methods by finding more high-quality *trans*-eQTLs
- *Trans*-PCO offers a map of *trans* regulation of gene networks and biological processes
- Functional annotation of gene modules helps functional interpretation of *trans* signals
- *Trans* effects via regulatory networks and pathways reveal the mechanism of trait loci

Authors

Lili Wang, Nikita Babushkin, Zhonghua Liu, Xuanyao Liu

Correspondence

xuanyao@uchicago.edu

In brief

Establishing a comprehensive map of *trans*-gene regulatory effects is a critical step toward understanding complex trait and disease genetics. However, detecting these effects is extremely difficult due to statistical and computational challenges. Wang et al. developed a powerful tool, *trans*-PCO, to detect high-quality *trans*-genetic effects on gene networks, which opens up new opportunities to learn the impact of trait-associated loci on gene regulatory networks.

Wang et al., 2024, Cell Genomics *4*, 100538
April 10, 2024 © 2024 The Author(s). Published by Elsevier Inc.
https://doi.org/10.1016/j.xgen.2024.100538

CellPress

Cell Genomics

CellPress
OPEN ACCESS

Article

Trans-eQTL mapping in gene sets identifies network effects of genetic variants

Lili Wang,[1,2] Nikita Babushkin,[2] Zhonghua Liu,[3] and Xuanyao Liu[1,2,4,5,*]

[1]The Committee on Genetics, Genomics and Systems Biology, University of Chicago, Chicago, IL 60637, USA
[2]Department of Medicine, Section of Genetic Medicine, University of Chicago, Chicago, IL 60637, USA
[3]Department of Biostatistics, Columbia University, New York, NY 10032, USA
[4]Department of Human Genetics, University of Chicago, Chicago, IL 60637, USA
[5]Lead contact
*Correspondence: xuanyao@uchicago.edu

https://doi.org/10.1016/j.xgen.2024.100538

SUMMARY

Nearly all trait-associated variants identified in genome-wide association studies (GWASs) are noncoding. The *cis* regulatory effects of these variants have been extensively characterized, but how they affect gene regulation in *trans* has been the subject of fewer studies because of the difficulty in detecting *trans*-expression quantitative loci (eQTLs). We developed *trans*-PCO for detecting *trans* effects of genetic variants on gene networks. Our simulations demonstrate that *trans*-PCO substantially outperforms existing *trans*-eQTL mapping methods. We applied *trans*-PCO to two gene expression datasets from whole blood, DGN (N = 913) and eQTLGen (N = 31,684), and identified 14,985 high-quality *trans*-eSNP-module pairs associated with 197 co-expression gene modules and biological processes. We performed colocalization analyses between GWAS loci of 46 complex traits and the *trans*-eQTLs. We demonstrated that the identified *trans* effects can help us understand how trait-associated variants affect gene regulatory networks and biological pathways.

INTRODUCTION

More than 90% of genome-wide association studies (GWASs) loci are located in noncoding regions of the genome and are thought to affect human traits by regulating gene expression.[1–5] Nearly all of the studies to date have focused on understanding the effects of trait-associated variants on gene expression in *cis*, which only include effects on genes that are near the associated loci. However, multiple lines of evidence suggest *cis*-regulatory effects capture only a small proportion of the heritability of complex traits and diseases. We previously hypothesized that *trans*-expression quantitative loci (eQTLs), despite having very small effects on each individual gene, may cumulatively account for a large proportion of trait variance.[6] Indeed, our modeling indicates that *trans*-eQTL effects account for twice as much genetic variance in complex traits as *cis*-eQTL effects.[6] Thus, establishing a representative map of genetic variants and their *trans* effects is a critical step toward understanding complex trait and disease genetics.

Two major challenges have precluded *trans*-eQTL discovery. First, *trans*-eQTL mapping is extremely prone to false positives due to mapping errors that cause short sequences to map to homologous regions of the genome.[7] The second challenge is by far more difficult to overcome: *trans*-eQTLs are challenging to detect compared to *cis*-eQTLs because (1) they have much smaller effect sizes than *cis*-eQTLs[6] and (2) a genome-wide search of *trans*-eQTLs involves a huge number of statistical tests, resulting in a heavy burden of multiple testing corrections.

Previous work suggests that *trans*-eQTLs generally affect the expression levels of multiple genes.[8,9] The co-regulation and co-expression patterns of genes driven by *trans*-eQTL have long been recognized. There are a few studies that aimed to identify *trans*-eQTLs of co-expressed genes. For example, Rotival et al.[10] used independent component analyses to identify co-expression gene sets, and subsequently tested for the enrichment of *trans* signals in the gene sets by hypergeometric tests. More recently, Kolberg et al.[11] tested associations between SNPs and an “eigengene” (essentially the primary principal component, PC1) of gene modules that captures the co-expression pattern. Nonetheless, PC1 has very limited power at identifying genetic effects (see below). Dutta et al.[12] leveraged canonical correlation analysis to identify *trans* associations between multiple disease-associated SNPs and multiple genes by integrating with GWAS signals. However, the method has different goals from identifying *trans*-eQTLs of multiple genes in specific tissues (e.g., it is useful for identifying “core”-like disease genes and processes for a specific disease; see below).

Our main goal was to develop a method for detecting *trans*-eQTLs associated with multiple genes in a gene module by using multivariate association. Multivariate association methods tend to be more powerful than univariate association methods. Detecting *trans*-eQTLs of gene modules containing multiple co-regulated genes can also potentially improve power by reducing multiple testing burdens, because the number of tested gene modules is much less than the number of genes. However, there are caveats. First, sequence similarity among distinct genomic

This is an open access article under the CC BY license (http://creativecommons.org/licenses/by/4.0/).

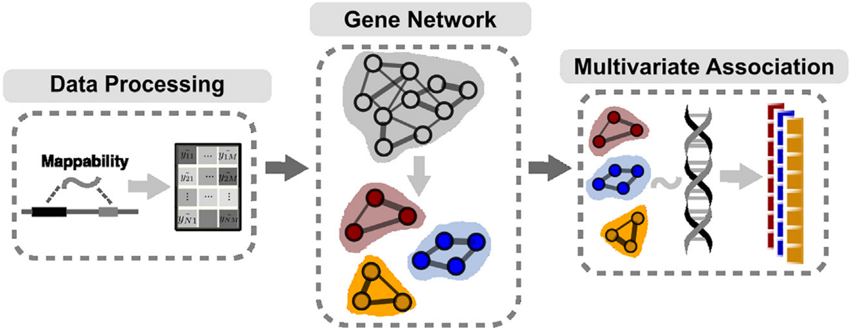

Figure 1. Three main steps of *trans*-PCO pipeline
First, *trans*-PCO preprocesses RNA-seq data to reduce false positive *trans*-eQTL associations. Second, genes are grouped into gene sets, such as co-expression modules or biological pathways. Lastly, *trans*-PCO tests the association between SNPs and gene sets by using PCO.

regions can lead to severe false positive discovery issues in *trans*-eQTL mapping.[7] This is especially problematic in mapping *trans*-eQTLs of co-expression gene modules because genes can be falsely clustered due to sequence similarities.[7,13] Second, the naive way of using a single component, such as the first gene expression PC, to represent the gene modules can significantly reduce power. Although the PC1 captures the largest amount of total variance in gene expressions, it can be powerless in detecting significant associations than higher-order PCs.[14,15]

To combat this, we propose *trans*-PCO, a flexible approach that uses the PC-based omnibus test[15] (PCO) to combine multiple PCs and improve power to detect *trans*-eQTLs. *Trans*-PCO also carefully filters sequencing reads and genes based on mappability across different regions of the genome to avoid false positives due to multimapping.[7,16,17] By default, *trans*-PCO uses gene sets identified by weighted gene co-expression network analysis (WGCNA),[18] which clusters co-expressed genes by using the correlations of gene expression levels. It also accepts user-defined sets—for example, genes that belong to the same Gene Ontology,[19] Kyoto Encyclopedia of Genes and Genomes pathway,[20] or protein complex.[21]

We applied *trans*-PCO to gene expression data from the Depression Genes and Networks study[16] (DGN, sample size N = 913) and the eQTLGen study[9] (sample size N = 31,684) to identify *trans*-eQTLs associated with co-expression gene modules and well-defined biological processes in whole blood. All *trans*-eQTLs that are associated with gene co-expression networks and biological pathways can be found at http://www.networks-liulab.org/transPCO.

RESULTS

Overview of the method

The *trans*-PCO method consists of three main steps (Figure 1). First, *trans*-PCO preprocesses RNA sequencing (RNA-seq) data to reduce false positive *trans*-eQTL associations due to read multimapping errors. Specifically, *trans*-PCO removes all of the sequencing reads mapped to low mappability regions of the genome (mappability score < 1; STAR Methods) before profiling gene expression levels. These procedures substantially reduce the occurrence of false positive *trans*-eQTLs due to sequencing alignment errors.[7,17] When only summary-level data are available (e.g., eQTLGen dataset[9]), *trans*-PCO dynamically excludes from the module any genes that are cross-mappable to genes within 100 kb of the tested SNP.

Second, *trans*-PCO groups genes into clusters. By default, *trans*-PCO determines the gene groupings by using WGCNA[18] to identify co-expression modules from gene expression levels (STAR Methods). We remove covariates and confounders (STAR Methods) from gene expression levels before grouping gene modules. This step is necessary to ensure that the gene modules are not primarily driven by confounding factors. *Trans*-PCO also allows customization of the gene groups or sets—for example, genes in the same pathway or protein-protein interaction network[19–21] can be grouped into user-defined gene modules.

Lastly, *trans*-PCO tests for association between each SNP and the expression levels of the genes in each gene module by adapting the PCO method, which combines multiple gene expression PCs by using six PC-based statistical tests (STAR Methods). Each PC-based test combines multiple PCs uniquely, which allow signals under various genetic architectures to be captured. PCO evaluates the six PC-based tests and takes the minimum p value as the final test statistic. The final p values are computed according to Liu and Lin[15] (also see STAR Methods). Only PCs with eigenvalues $\lambda_k > 0.1$ are used in *trans*-PCO (Figure S1; Methods S1). To avoid identifying associations driven by *cis* effects, we excluded from the module all of the genes on the same chromosome as the test SNP. To correct for multiple testing, we performed 10 permutations to establish an empirical null distribution of p values (Methods S1).

Trans-PCO outperforms existing methods in simulations

We performed simulations to evaluate the power of *trans*-PCO in detecting *trans*-eQTLs associated with multiple genes. We primarily compared the power to (1) the standard univariate test ("MinP") and (2) the PC1-based test (Kolberg et al.[11]; STAR Methods). We used a co-expression gene module consisting of 101 genes from the DGN dataset (module 29). In power simulations, we simulated a proportion of 101 genes in the module to be causal with nonzero effects generated from a point normal distribution (STAR Methods). We simulated the *trans* genetic variance to be 0.001, which is a low and realistic per SNP heritability for *trans* effects. In null simulations, all SNPs effects have the same *trans* genetic variance but zero average effects (STAR Methods).

Trans-PCO significantly outperformed the univariate test and the PC1 method across different sample sizes and proportions of causal genes (Figure 2). Specifically, the power of *trans*-PCO increases rapidly with increasing sample sizes. At a sample size of 800, assuming 30% of genes have causal effects in the

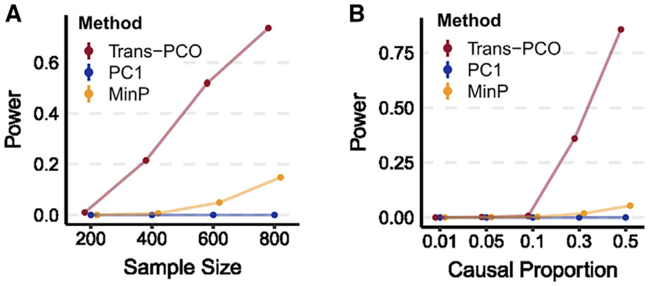

Figure 2. Power of *trans*-PCO across different sample sizes and causal gene proportions, in comparison to PC1 and univariate (MinP) methods

Points show average power across 1,000 simulations. Error bars representing 95% confidence intervals (CIs) are plotted, but many are too small to be visible. See numerical results in Table S2.

(A) Power comparison across various sample sizes. *Trans*-genetic variance was simulated to be 0.001, and the proportion of causal genes in the gene module was 30%.

(B) Power comparison across different proportions of causal genes in the gene module. The simulated sample size was 500.

gene module, the power of *trans*-PCO is 74%, compared to 15% for the univariate test and 0.0018% for the PC1 method (Figure 2A).

We also compared the power of each method across various causal gene proportions using a fixed sample size (500). All 3 methods have little power in detecting *trans*-eQTLs when the proportion of causal genes is below 10%. However, above this threshold, the power of *trans*-PCO increases dramatically: 36% at 30% causal genes and 86% at 50% causal genes. In contrast, the univariate and the PC1 methods remain almost powerless for nearly all of the simulated scenarios (Figure 2B). We note that the PC1 method appears to be almost powerless across the scenarios, which agrees with the previous observation that the PC1 can be less powerful than higher-order PCs in GWASs.[22] Simulation results at various genetic variances can be found in the supplemental information, including at extremely low proportions of causal genes and high *trans* effects (Figure S2). We found that the univariate method only outperforms *trans*-PCO when the proportion of causal genes is extremely low, such as only one causal gene in the entire gene set, and the *trans* effects are large. *Trans*-PCO gains more power when there are >1 causal gene because it aggregates multiple weak effects to improve power. Null simulations demonstrated that all three methods are well controlled for false positive inflations (Figure S3).

We included comparisons to two additional methods: ARCHIE, proposed by Dutta et al.,[12] and a method by Rotival et al.[10] (Figures S4 and S5; Methods S2). We showed that ARCHIE is not powerful at detecting *trans*-eQTL effects from a SNP to multiple genes, which are the effects for which *trans*-PCO was designed (Figure S4). We note that the main goal of ARCHIE is to identify trait-specific gene sets associated with GWAS loci, whereas *trans*-PCO is designed to map *trans*-eQTLs for any user-specified gene sets in specific tissues or cell types (discussion; Figure S4; Methods S2). The method of Rotival et al.[10] is based on the PC1-based approach, and we showed that the method has limited power at identifying weak *trans*-eQTL effects (Figure S5; Methods S2).

Trans-PCO identifies 3,899 *trans*-eSNP-module pairs associated with co-expression gene modules in the DGN dataset

We used *trans*-PCO to identify *trans*-eQTLs associated with co-expression gene modules in RNA-seq data from whole-blood samples of the DGN cohort (N = 913).[16] WGCNA[18] identified 166 co-expression gene modules, with the number of genes in each module ranging between 625 (module 1 [M1]) and 10 (M166) (Table S1). We then performed genome-wide scans of *trans*-eQTLs for each gene module. At a 10% false discovery rate (FDR), *trans*-PCO identified significant *trans*-eQTLs for 102 of 166 gene modules, corresponding to 3,899 significant *trans*-eSNP-module pairs (Table S3). Many *trans*-eSNPs are in linkage disequilibrium (LD). Using LD clumping to group *trans*-eSNPs into LD-independent loci ($R^2 < 0.2$), we found 202 *trans*-loci-module pairs (Figures 3A and S6; Tables S3 and S4).

We compared *trans*-eQTL signals detected in DGN by *trans*-PCO to signals identified by the univariate method in Battle et al.[16] Of the 12,132 genes analyzed by *trans*-PCO, the univariate method detected 326 significant *trans*-eSNP-gene pairs for 128 genes at 5% FDR.[16] At the same FDR level, *trans*-PCO identified 3,031 significant *trans*-eSNP-gene module pairs for 75 gene modules. We compared the magnitude of the significant *trans*-eQTL effects detected by *trans*-PCO and the univariate method. More specifically, we compared the maximum univariate Z scores of SNPs and each gene in significant *trans*-eSNP-module pairs identified by *trans*-PCO to the Z scores of significant *trans*-eSNP-gene pairs by the univariate method. We found that the maximum Z scores of *trans*-PCO signals are much smaller than Z scores of the univariate method signals (Figure 3B), indicating that our multivariate approach can detect much smaller *trans* effects than univariate methods.

We also applied the PC1 method (Kolberg et al.[11]) to DGN and identified 1,483 significant *trans*-eSNP-module pairs (55 *trans*-loci-module pairs) at 10% FDR, and 1,464 pairs (99%) were detected by *trans*-PCO (Figure S7A). Notably, in total, *trans*-PCO identified more than twice the signals of the PC1 method. However, the PC1 method identified more signals than expected because it was previously shown to be powerless in the simulations. We note that we simulated weak effects and sparse causal proportions to better reflect common and realistic *trans* effects, and the PC1 method is powerless in these settings. We performed additional simulations with large effects and high causal proportions, and effects with aligned direction of the PC1,[15] and the PC1 method achieved 50% power as *trans*-PCO or even the best power (Figures S8 and S9; Methods S2). In addition, we found in the DGN dataset that the univariate Z scores of *trans* signals detected by the PC1 method are larger than those of *trans*-PCO signals (Figures S7B–S7D). Therefore, the *trans* signals detected by the PC1 method are likely of strong *trans* effects, and *trans*-PCO is able to detect additional weak *trans* effects.

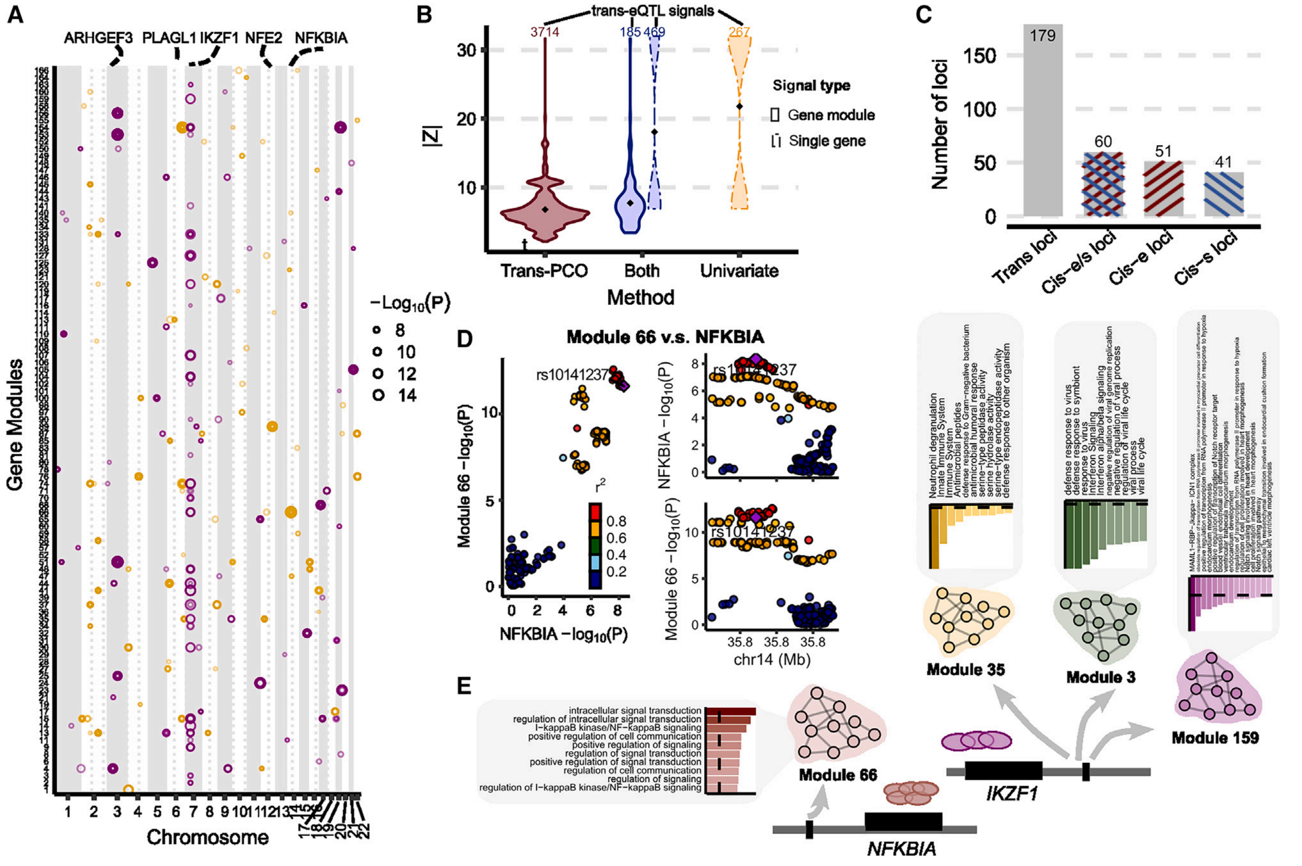

Figure 3. *Trans*-PCO identifies *trans*-eQTLs associated with co-expression gene modules in DGN

(A) Significant *trans*-eQTL signals associated with 166 co-expression modules in DGN. Chromosomal positions of *trans*-eSNPs are on the x axis, and gene modules are on the y axis. Point sizes are $-\log_{10}(p)$ values of significant *trans*-eQTLs. Purple and orange represent odd and even chromosomes, respectively.

(B) Comparison of the magnitude of significant *trans*-eQTLs effects detected by *trans*-PCO and the univariate method. The x axis shows signal categories: *trans*-PCO specific signals (*Trans*-PCO), univariate test specific signals (Univariate), and signals identified by both methods (Both). The maximum Z scores of each SNP and each gene in a gene module is used to represent the SNP-module pair. The numbers on top are the number of signals in each category. Line type represents the target type of signals (gene module vs. single gene). The y axis is the absolute value of the Z scores of the signals.

(C) Colocalization of *trans*-eQTLs and *cis*-e/sQTLs. The gray bar represents the *trans*-region used for colocalization analyses. The bar highlighted in blue represents the *trans*-region colocalized with *cis*-sQTLs, red for *cis*-eQTLs, and mixed color for either *cis*-eQTLs or *cis*-sQTLs.

(D) Colocalization of *trans*-eQTLs of M66 and *cis*-eQTLs of *NFKBIA*.

(E) Functional annotations of gene sets facilitate functional interpretation of *trans*-eQTL signals. The *trans*-eQTLs near *NFKBIA* and *IKZF1* are associated with several gene modules. The bar plots show the functional enrichments in modules. The numerical values of enrichments are in Table S7.

***Trans*-eQTLs are enriched in variants with *cis*-regulatory effects on transcription factors**

We found that only 31 *trans*-eSNPs (1%) are in coding regions, suggesting that a very small proportion of *trans*-eQTLs affect gene expression levels in *trans* by altering protein coding sequences. Several studies have shown that *trans*-eQTLs have *cis*-regulatory effects, affecting the expression levels or splicing of nearby genes[9,16]; thus, we evaluated our identified *trans*-eQTLs for concomitant *cis*-regulatory activity. We first overlapped *trans*-eSNPs with *cis*-eQTLs and *cis*-splicing QTLs (*cis*-sQTLs) in DGN.[23] Of the 2,955 *trans*-eSNPs (Table S3), we found that 71% are significant *cis*-eSNPs in DGN and 46% are significant *cis*-sSNPs, together accounting for 73% of all *trans*-eSNPs. To further examine whether the *cis* and *trans* effects are driven by the same variant, we performed colocalization analysis of *trans*-eQTLs with *cis*-eQTLs and *cis*-sQTLs using coloc[24] (STAR Methods). Specifically, we first grouped *trans*-eSNP-gene module pairs into 179 *trans*-region-gene module pairs, based on 200-kb fixed-width regions (STAR Methods). We then performed colocalization analyses between the *trans*-eQTLs and *cis*-eQTLs/*cis*-sQTLs. We found that 51 of 179 *trans* regions colocalized with a *cis*-eQTL (the posterior probability of colocalized signals PP4 > 0.75; Figures 3C and S10). A total of 41 *trans*-regions colocalized with a *cis*-sQTL. Overall, 60 *trans*-regions shared causal variants with at least one *cis*-eQTL or *cis*-sQTL (Figure 3C; Table S5), confirming that *trans*-eQTL effects are generally mediated through *cis*-gene regulation. In addition, a large fraction of *trans*-loci (66%) do not colocalize with *cis*-eQTLs or *cis*-sQTLs. Although power may have limited our ability to detect colocalization of some *trans*-eQTLs and *cis*-eQTLs, there may also exist unknown *trans*-regulatory mechanisms, independent of *cis*-gene expression or splicing, which is subject to future studies.

CellPress
OPEN ACCESS

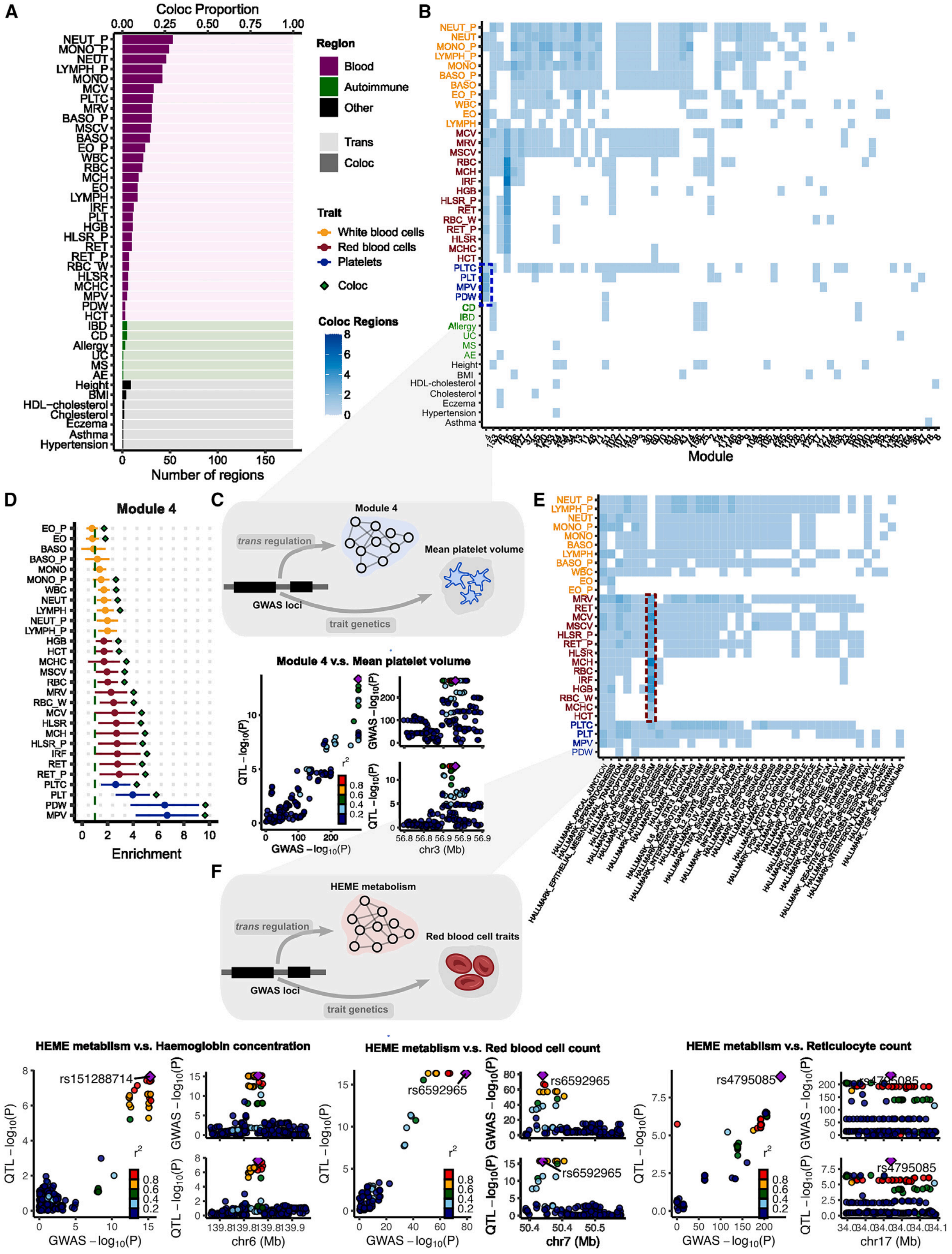

(legend on next page)

We also investigated the types and functions of genes that are likely to mediate *trans*-eQTL effects. We found that the genes nearest *trans*-eQTLs are highly enriched in "RNA polymerase II transcription regulatory region sequence-specific DNA binding" (adjusted p = 1.26×10^{-3}) and "DNA-binding transcription factor activity" (adjusted p = 1.39×10^{-3}; Table S6), suggesting that transcription factors are important mediators of *trans*-eQTL effects. Indeed, *trans*-PCO identified and replicated several well-known master *trans* regulators in blood, such as *IKZF1*,[17,25,26] *NFKBIA*,[17] *NFE2*,[9,17,27] and *PLAGL1*[17,26] (Figure 3A). We also found colocalization of these *trans*-eQTLs with *cis*-eQTLs at the *NFKBIA*, *NFE2*, and *PLAGL1* loci (Figures 3D and S10), supporting the conclusion that these genes are likely the *cis*-mediating genes.

High-quality map of *trans*-eSNP to gene module associations improves functional interpretation

Most of the gene modules used in *trans*-PCO have functional annotations, which allows us to interpret the functional roles of the *trans*-eQTLs identified by the method. We first functionally annotated the 166 co-expression modules using g:Profiler,[28] which performs functional enrichment analysis on gene sets using predefined Gene Ontology and pathway annotations. This allowed us to annotate 131 of the 166 modules with at least 1 significantly enriched Gene Ontology or pathway (Table S7).

These annotations helped us interpret the function of identified *trans* effects. For example, the *trans*-eQTL signal near *IKZF1* (on chromosome 7) is significantly associated with 27 gene modules. *IKZF1* encodes a transcription factor, IKAROS, that belongs to the family of zinc finger DNA-binding proteins.[29] The *IKZF1* (IKAROS) *trans*-target gene M159 is significantly enriched in the "positive regulation of transcription of Notch receptor target" (adjusted p = 6.82×10^{-3}; Figure 3E). We were reassured to find that it previously had been found that IKAROS is a repressor of many Notch targets, and our *trans*-eQTL signal further supports the *trans* regulation of Notch signaling pathway by IKAROS.[30] *IKZF1 trans*-target M3 is significantly enriched in the Gene Ontology term "defense response to virus" (Figure S11; adjusted p = 8.7×10^{-31}), and M35 is significantly enriched in the innate immune system (adjusted p = 4.09×10^{-17}). These data support the conclusion that the *IKZF1* locus plays a *trans*-regulatory role in immune responses (Figure 3E). The *trans*-eQTLs near *NFKBIA*, which encode nuclear factor (NF)-κB inhibitor subunit A, are significantly associated with M66 ($p < 1.8 \times 10^{-7}$). Interestingly, we found that M66 is highly enriched in NF-κB signaling pathway (adjusted p = 8.35×10^{-5}; Figure 3E), which supports the *trans*-regulation of the NF-κB signaling pathway by *NFKBIA*. The complete list of *trans*-eQTLs signals and functional annotations of *trans*-target gene modules can be found in Tables S4 and S7.

Trans-PCO identifies 965 *trans*-eSNP-module pairs associated with well-defined biological processes

To further demonstrate the utility of *trans*-PCO, we applied *trans*-PCO to 50 Human Molecular Signatures Database (MSigDB) hallmark gene sets, which represent well-defined biological processes,[19] including DNA repair, coagulation, heme metabolism, and Notch signaling (Table S8). Each gene set contains between 32 and 200 genes. In DGN, we identified 965 significant *trans*-eSNP-module pairs, corresponding to 41 gene sets and 120 *trans*-loci-module pairs ($R^2 < 0.2$), at a 10% FDR level (Figure S12; Tables S3 and S9).

Trans-eQTLs associated with well-defined biological processes facilitate the interpretation of the *trans*-eQTL signals. For example, we identified several *trans*-eQTL signals at the *NLRC5* locus (Table S9). The *trans*-target gene set is the "interferon alpha response" gene set, suggesting *trans* regulation from *NLRC5* to the interferon signaling pathway. Earlier studies have confirmed that *NLRC5* is a master regulator for major histocompatibility complex (MHC) class II genes and negatively regulates the interferon signaling pathway.[31,32] The *trans*-eQTL signals also validated our previous interpretations of *trans*-eQTLs associated with co-expression gene modules. For example, in agreement with our analysis of co-expression modules, we found that the *IKZF1* locus is significantly associated with several immune-related biological processes, such as interferon-gamma response (Figure 3E; Table S9).

Trans-PCO improves understanding of *trans*-regulatory effects of disease-associated loci

To understand the *trans*-regulatory effects of genetic variants associated with complex traits, we performed colocalization analysis of *trans*-eQTL signals with GWAS loci of 46 complex traits and diseases, including 29 blood traits and 8 other common complex traits (e.g., height, body mass index) from the UK Biobank,[27,33] provided by Neale Lab (http://www.nealelab.is/uk-biobank/), and 9 autoimmune diseases[23,34–40] (Table S10; STAR Methods).

We grouped the *trans*-eSNPs into 200-kb regions (or *trans*-regions) for colocalization analyses (STAR Methods). The 3,899 *trans*-eQTLs associated with co-expression gene modules were grouped into 179 *trans*-region-module pairs. Of the 46 complex traits, 42 have at least 1 GWAS loci colocalized with 1 of 179 *trans*-region-module pairs. On average across all of the traits, 8.8% of *trans*-loci colocalize with GWAS loci

Figure 4. Colocalization of *trans*-eQTLs with GWAS loci of 42 complex traits with at least 1 colocalization region
(A) The number of colocalized *trans*-regions associated with co-expression gene modules with GWAS loci. The proportion of colocalization is the proportion of colocalized *trans*-loci over 179 *trans*-regions.
(B) Heatmap of the number of colocalized *trans*-regions associated with co-expression gene modules with GWAS loci between each module and trait. Tiles represent the number of colocalized regions.
(C) Colocalization of mean platelet volume-associated locus near *ARHGEF3* and *trans*-eQTL of M4.
(D) Heritability enrichment of M4 in blood traits. Error bars are 95% CIs.
(E) Heatmap of the number of colocalized *trans*-regions associated with MSigDB hallmark gene sets with GWAS loci.
(F) Colocalization of GWAS loci-associated red blood cell traits and *trans*-eQTLs associated with heme metabolism. Six loci associated with red blood cell traits are associated with heme metabolism in *trans*. Numerical results can be found in Table S14. Colocalization plots of the other loci are in Figure S13.

(Figure 4A; Table S11). We observed a higher proportion of colocalization with blood traits (mean proportion 12.0%) than non-blood traits (mean proportion 1.5%). Although we expect some higher proportions of colocalization with blood traits to occur in a whole-blood sample, our results may also indicate some residual effects due to cell composition, despite corrections for cell composition using both gene expression PCs and estimated cell-type proportions,[16] such that some *trans*-eQTLs may regulate the abundance of cell proportions and therefore are associated genes that are specifically expressed in certain cell types (discussion). Our results are consistent with a recent study by the eQTLGen consortium, which has shown that *trans*-eQTLs in whole blood reflect a combination of cell-type composition and intracellular effects.[9]

Nevertheless, we found several *trans*-eQTLs that colocalized with GWAS loci, which revealed specific interpretable pathways or functional gene sets (Figure 4B; Table S12). For example, *trans*-eQTLs associated with co-expression M4 colocalized with 24 of 29 blood traits (Figure 4B). M4 is highly enriched for genes involved in platelet activation (adjusted $p = 1.12 \times 10^{-12}$; Figure S11; Table S7). One of the colocalized *trans*-eSNPs associated with M4 is in the introns of the *ARHGEF3* gene (Figure 4C), which has been shown to play a significant role in platelet size in mice.[41] To further support the interpretation of colocalized signals, we estimated heritability enrichment of M4 in blood traits using stratified LDscore regression[42] (S-LDSC; Figures 4D and S13). We reasoned that an enrichment of trait heritability near genes in a module would strongly support the involvement of a module in the genetic etiology of a trait. Strikingly, we found that M4 is significantly enriched in the heritability of multiple blood traits, and that the enrichment was especially strong for platelet traits such as platelet distribution width (odds ratio [OR] = 6.5, $p = 7.0 \times 10^{-5}$) and mean platelet volume (OR = 6.7, $p = 1.2 \times 10^{-5}$; Figure 4D; Table S13). In addition, we evaluated whether M4 genes are significantly enriched in genes associated with platelet traits, identified by transcriptome-wide association studies (TWASs). There are 1,339 unique genes significantly associated with platelet traits in the UK Biobank.[43] M4 genes are significantly enriched in TWAS genes associated with platelet traits (88 overlap genes, $p = 6.7 \times 10^{-10}$, Fisher's exact test), which further supports the role of M4 in platelet traits. Finally, we identified that the *ARHGEF3* locus is significantly associated with the MSigDB coagulation hallmark gene set (Table S9). These findings strengthen the model in which genetic variation near *ARHGEF3* affects the expression levels of multiple genes that are involved in platelet biology and that also harbor nearby genetic variation associated with platelet traits.

We also performed colocalization analysis of *trans*-eQTLs-associated MSigDB hallmark gene sets (Figure 4E; Table S14). One of the gene sets represents heme metabolism, which is an essential process underlying erythroblast differentiation and red blood cell counts. We found that six *trans*-eQTL loci of heme metabolism significantly colocalized with GWAS loci associated with red blood cell traits, such as hemoglobin concentration, red blood cell count, and reticulocyte count (PP4 = 0.76–1.00; Figures 4F and S14; Table S14). We found that the genes in the gene sets are significantly enriched in TWAS-significant genes associated with hemoglobin levels in the UK Biobank (35 overlap genes, $p = 8.1 \times 10^{-4}$, Fisher's exact test), which further supports the role of the hallmark gene set in red blood cell traits. Our results provide evidence that these six loci regulate heme metabolism in *trans,* which is an essential process underlying erythroblast differentiation and red blood cell counts.

In another example, we found a *trans*-eQTL near *IKZF1* for M3 that colocalizes with 11 blood traits, 7 of which are related to white blood cells (Table S12). As mentioned previously, M3 is significantly enriched for Gene Ontology terms, including "defense response to virus" (adjusted $p = 8.7 \times 10^{-31}$) and "negative regulation of viral processes" (adjusted $p = 1.07 \times 10^{-17}$; Table S7). The enrichments are driven by many genes related to interferon (e.g., *IFI6*, *IFI16*, *IRF7*), which are proteins released by host cells in response to the presence of viruses and indicate immune-related functions (Tables S1 and S7). In addition, our heritability analysis of genes in M3 identified enrichments for multiple traits associated with blood cell-type count, including neutrophil count (OR = 2.3, $p = 1.7 \times 10^{-4}$) and white blood cell count (OR = 2.1, $p = 1.3 \times 10^{-4}$, Figure S15). Our analyses support that the white blood cell associated locus *IKZF1* regulates immune-response pathways in *trans*.

Taken together, our functional map of *trans*-eQTLs revealed concrete examples where genetic variants associated with complex traits also influence a biological pathway or a coherent set of genes with similar functions. Thus, *trans*-eQTL of gene sets have the potential to reveal *trans*-regulatory mechanisms underlying complex traits and diseases. The complete list of colocalization signals for each trait can be found in Table S12.

Summary statistics-based *trans*-PCO identified 10,167 *trans*-eSNP-module pairs in eQTLGen

We developed summary statistics-based *trans*-PCO to increase its applicability to gene expression datasets of large sample sizes, such as eQTLGen[9] (N = 31,684, whole blood). To ensure that summary statistics-based *trans*-PCO signals are well controlled for test statistics inflation and false positives, we added two steps to the original pipeline. First, we carefully selected gene sets to minimize the noise when approximating the gene correlation matrices. When only summary statistics are available, the correlation matrix of each gene set is approximated with the correlations of Z scores of the insignificantly associated SNPs of each gene. A low ratio of SNPs to genes (<50) results in a noisy approximation of correlation matrices and test statistics inflation (Figure 5A; STAR Methods; Methods S3). Therefore, we only used gene modules with ratios >50 to test for *trans*-eQTLs, which we show are well controlled for inflation (Figures 5A and S16). Second, we removed genes in the module that were cross-mappable to the test SNP loci (STAR Methods) in the association test to reduce false positives caused by multimapping reads.

The eQTLGen study performed the standard univariate *trans*-eQTL mapping on a subset of 10,317 GWAS SNPs, and the summary statistics of these *trans*-eQTLs are available. We applied the summary statistics-based *trans*-PCO to these summary statistics to identify *trans*-eQTLs-associated co-expression gene modules and MSigDB hallmark gene sets.

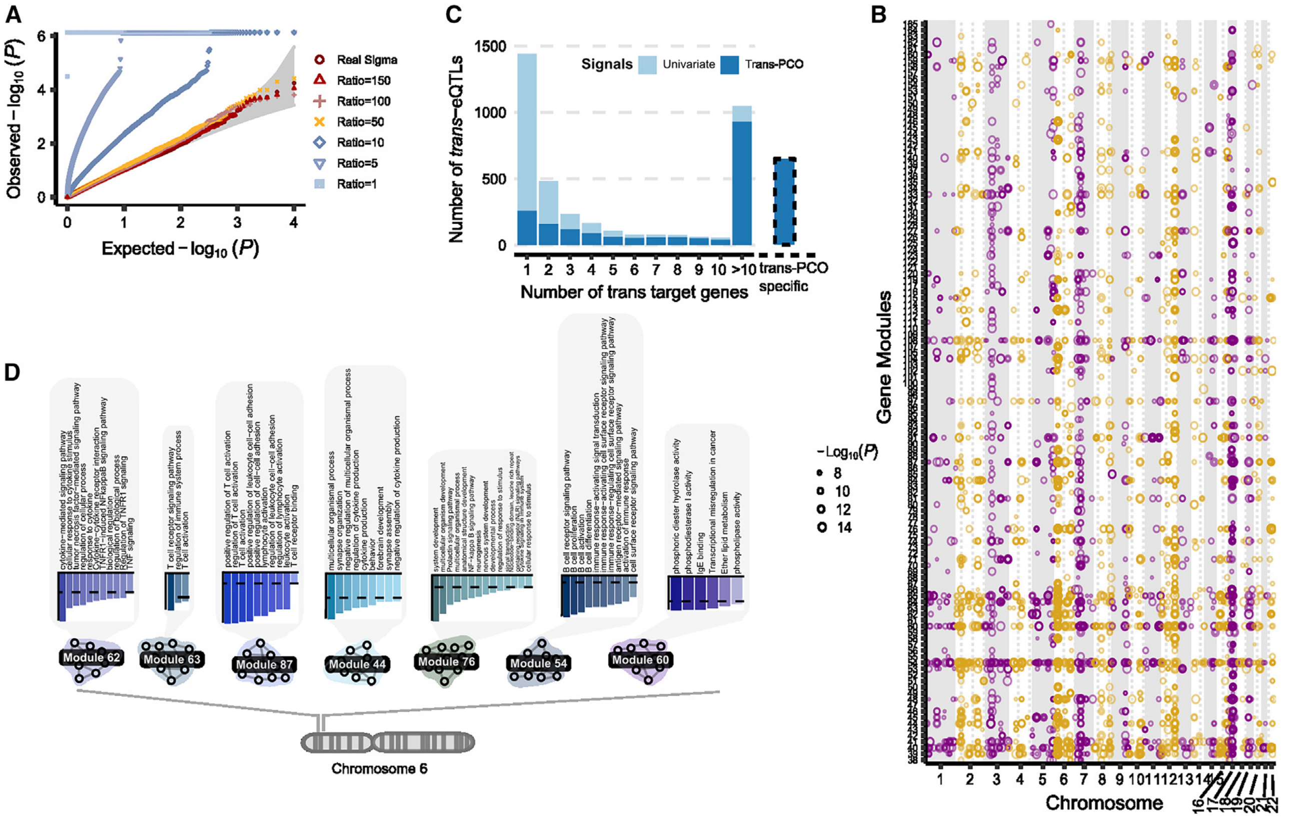

Figure 5. *Trans*-PCO identifies *trans*-eQTLs associated with co-expression gene modules and MSigDB hallmark gene sets in eQTLGen

(A) Summary statistics-based *trans*-PCO is well controlled for test statistics inflations. We show gene module 1 (size 625) as an example. SNP-to-gene ratios used for correlation matrix estimation are in different shapes and colors. Red-yellow shades represent higher ratios (≥ 50), and blue shades represent lower ratios. Gray area shows 95% CIs. *Trans*-PCO used a minimum ratio of 50.

(B) 8,199 significant *trans*-eSNP-module pairs associated with co-expression modules in eQTLGen. Chromosomal positions of *trans*-eSNPs are on the x axis and gene modules are on the y axis. Point sizes are $-\log_{10}(p)$ values of significant *trans*-eQTLs.

(C) The majority of hub SNPs targeting >10 genes in the original eQTLGen study are identified by *trans*-PCO. The light blue bar represents the total number of *trans*-eQTLs in the original eQTLGen study at 5% FDR level. The dark blue bar represents the *trans*-eQTLs also detected by *trans*-PCO under Bonferroni correction that are associated with co-expression modules or MSigDB gene sets. The bar at right shows the *trans*-eQTLs detected only by *trans*-PCO.

(D) The HLA locus is associated with several immune-related gene modules in *trans.* The bar plots show the functional enrichment of co-expression gene modules.

Of the 166 co-expression gene modules identified in DGN, we used 129 modules with reliable correlation matrix approximations to ensure that the *trans*-eQTL signals were well controlled for inflation (Figures 5A and S16; STAR Methods; Methods S3). Similarly, of the 50 MSigDB hallmark gene sets, we only used 11 gene sets with accurate correlation matrix approximations (Figure S17). In total, there were 4,533 genes in the tested co-expression gene modules and hallmark gene sets. For co-expression gene modules, we identified 8,116 *trans*-eSNP-gene co-expression module pairs, corresponding to 2,161 eQTLGen test SNPs and 122 gene modules (Figure 5B; Tables S3 and S15). For hallmark gene sets, we found 2,051 significant *trans*-eSNP-hallmark gene set pairs, corresponding to 1,018 SNPs and all 11 hallmark gene sets, using Bonferroni correction (Tables S3 and S16). In eQTLGen, we did not perform LD clumping on *trans*-eSNPs because they were GWAS SNPs associated with different traits and diseases. The univariate method used in eQTLGen[9] identified 1,050 hub SNPs targeting >10 genes at 5% FDR, 89% of which were also identified by *trans*-PCO (Figure 5C).

The large sample size in eQTLGen improves the power of *trans*-eQTL detection. Of the 3,899 significant *trans*-eSNP-co-expression module pairs in DGN, 38 pairs were also tested in eQTLGen. We did find that all 38 *trans* signals were replicated in eQTLGen (under a replication p value cutoff of 0.1/38; Table S17) and all association p values were highly significant ($p < 10^{-12}$; Figure S18). In contrast, most of the *trans*-eQTL signals in eQTLGen were not found in DGN. For example, of the 7,577 SNP-module pairs analyzed in both datasets, there were 7,291 pairs (96%) that were uniquely identified in eQTLGen (defined as at least 1 MB away from *trans*-eQTL SNPs in DGN). This is not surprising because the association p values are much smaller in the eQTLGen dataset due to the larger sample size (Figure S19). Similarly, 8 significant *trans*-eSNP–hallmark gene set pairs in DGN were tested in eQTLGen, and all of them were replicated. We also compared eQTLGen signals by

CellPress
OPEN ACCESS

trans-PCO to those identified by ARCHIE in Dutta et al.[12] (Figure S4; Methods S2).

The nearest genes of eQTLGen *trans*-eQTLs are significantly enriched in DNA-binding activity (adjusted p = 3.73×10^{-4}) and transcription factor binding (adjusted p = 1.74×10^{-7}), as well as immune responses such as cytokine receptor activity (adjusted p = 7.27×10^{-7}) or MHC class II receptor activity (adjusted p = 9.93×10^{-5}; Figure 5B; Table S18). We found that the enrichment of immune responses was driven by *trans*-eQTLs in the human leukocyte antigen (HLA) region on chromosome 6 (e.g., *HLA-DRA*, *HLA-DRB1*; Table S15) or near cytokine receptor genes (e.g., *IL23R*, *IL1R1*, *CXCR4*; genes on the chemokine receptor gene cluster region*: CCR2*, *CCR3*, *CCR5*, etc.). These *trans*-eQTLs are associated with several autoimmune diseases, such as type 1 diabetes, autoimmune thyroid diseases, cutaneous lupus erythematosus, and inflammatory bowel disease (Table S15). The *trans*-PCO signals help us understand the *trans*-regulatory mechanism of these loci. For example, we found that the *trans*-target gene modules of the HLA loci are enriched in immune-related functions, such as cytokine production (M44), B cell differentiation (M54), immunoglobulin E (IgE) binding (M60), tumor necrosis factor signaling pathway (M62), T cell activation (M63 and M87), and cytokine signaling pathway (M62 and M76; Figure 5D). The *IL23R* locus is associated with cytokine signaling pathway (M76) in *trans*. The chemokine receptor genes were associated with several gene modules, including cytokine production (M44), IgE binding (M60), and T cell activation (M87). These *trans*-eQTL signals support the conclusion that genetic loci associated with autoimmune disease regulate immune-related pathways in *trans*.

DISCUSSION

In summary, we developed a powerful method, *trans*-PCO, to detect *trans*-eQTLs associated with expression levels of co-expressed or co-regulated genes. The multivariate approach of *trans*-PCO can detect much smaller *trans* effects and is substantially more powerful than existing methods.

We thoroughly compared the performance of *trans*-PCO versus other methods, such as the PC1-based method by Kolberg et al.,[11] ARCHIE by Dutta et al.,[12] and Rotival et al.[10] (Figures 2, S4, S5, and S7–S9; Methods S2). *Trans*-PCO- and the PC1-based method are both designed to identify individual *trans*-eQTLs of any gene sets containing multiple genes, and the comparison between them is straightforward. However, ARCHIE is different and not directly comparable to the other two methods for several reasons (see more discussions in Methods S2). First, ARCHIE captures only trait-specific *trans*-regulations, by testing significance against a null hypothesis based on a subset of genetic variants that are trait associated. In contrast, *trans*-PCO identifies *trans*-eQTLs under the general null hypothesis with no additional assumptions. Second, *trans*-PCO and ARCHIE are designed to capture different *trans*-regulatory effects. ARCHIE is powerful when multiple disease-associated variants have weak effects on a single gene or multiple disease-associated variants have weak effects on multiple genes (Figure 2 in Dutta et al.[12]), which are not co-regulated by a shared *trans* genetic locus. In contrast, *trans*-PCO is designed to capture weak *trans* signals of a variant on multiple co-regulated genes (Figure S4; Methods S2). Third, ARCHIE identifies components consisting of multiple trait-associated SNPs and multiple genes, without knowing the exact *trans*-eQTL SNP driving the *trans*-regulation. It is hard to further study *trans*-regulatory mechanisms of the *trans*-eQTLs. Fourth, ARCHIE takes all of the genes as input and infers gene sets that are *trans*-regulated by disease-associated variants, whereas *trans*-PCO is flexible to be applied to any user-defined gene set of interest to identify *trans*-eQTLs. In summary, *trans*-PCO and ARCHIE have different goals and are designed for detecting different types of *trans* signals. However, we thoroughly compared ARCHIE and *trans*-PCO in both simulations and real data analyses (Methods S2). We believe that these comparisons will provide insights into when and how these methods should best be used.

Trans-eQTLs identified in bulk tissues can be a combination of cell composition *trans*-eQTLs, which are driven by cell-type proportions, and intracellular *trans*-eQTLs, which capture *trans*-regulatory effects in a single cell type. In our analysis of DGN dataset, we included the estimated cell proportions as covariates, in addition to gene expression PCs, to obtain higher proportions of intracellular *trans*-eQTLs. Co-expression gene modules could also capture cell proportion effects. In our study, we removed cell proportions from gene expression levels before clustering genes into co-expression modules. Although this can correct for cell proportion effects in the co-expression modules to some extent, we note that it does not guarantee their complete removal.

Many studies, including ours, seek to avoid cell composition effects. However, by closely examining *trans*-eQTLs discovered in our study, we think that cell composition *trans*-eQTLs can also be biologically interesting. For example, the *IKZF1* locus is significantly associated with several gene modules enriched with viral defense and other immune-related functions in *trans*. The locus is also significantly associated with white blood cell proportions. Given the general function of white blood cells in fighting infections, these observations raise the possibility that the *trans*-eQTLs near *IKZF1* regulate antiviral activity by affecting white blood cell-type proportion. Supporting this hypothesis, we found earlier that genetic variants near *IKZF1* are also associated with expression levels of genes in M159, which are enriched in genes involved in the Notch signaling pathway. The Notch signaling pathway plays a central role in cell proliferation, cell fate, and cell differentiation[44]; thus, our analyses reveal a plausible mode of action whereby genetic variants near *IKZF1* affect multiple immune-related functions by influencing white blood cell-type proportions.

Identifying the network effects of genetic variants not only shed light on molecular mechanisms of complex associated loci, but it can also have important translational applications. First, genes that are associated with disease-relevant pathways can serve as evidence for therapeutic targets of the disease. In a preliminary analysis, we examined whether allergy drug targets are more likely to be associated with immune-related gene sets. Among a total of 142 gene sets used for *trans*-eQTL identification in eQTLGen, 19 were defined as immune related. We used 55 launched allergy drug target genes from the Broad Institute Drug Repurposing Hub

(https://repo-hub.broadinstitute.org/repurposing), 5 of which are near allergy-associated loci in eQTLGen. Interestingly, we found all 5 targets to be associated with immune-related gene sets (Table S19). Detailed analyses can be found in STAR Methods and Methods S4. Although the enrichment is not statistically significant (p = 0.12, Fisher's exact test; Table S20), it is likely due to the small number of drug targets included in our analyses. In addition, we observed that the *trans*-gene modules of drug targets converge to gene sets whose functions are highly relevant to allergy. For example, three drug targets (*IL3*, *UGT3A1*, and *SLC37A4*) are associated with gene sets enriched for the B cell signaling pathway. Second, network effects of disease variants can be used for repurposing existing drug compounds to new diseases. Drug repurposing can substantially reduce cost and time to develop new treatments. If the gene expression profile of an existing drug is enriched for genes in the *trans*-network of associated loci of another disease, it can serve as evidence for repurposing. We believe comprehensive catalogs of *trans*-networks effects in human cell types and tissues will serve as important resources for the interpretation of *trans*-regulatory effects of disease-associated loci as well as translation applications. Therefore, we made all of the *trans*-PCO *trans*-eQTL signals, with functional annotation of the gene sets, publicly available, downloadable, and browsable in http://www.networks-liulab.org/transPCO.

Limitations of the study

A limitation of multivariate association tests, including *trans*-PCO, is that they do not explicitly identify which genes in the gene sets are significantly associated with the test SNP. Although functional annotations of gene sets facilitate our understanding of the *trans*-eQTL signals, it is possible that the genes driving *trans* associations are different from the genes driving functional enrichment of the gene sets. Therefore, the biological interpretation of *trans*-eQTL signals should be supported with other evidence before it is considered definitive. However, there are exploratory analyses that can help prioritize genes in the network that are key drivers of the underlying signal. For example, by examining the univariate association p values between the *trans*-eQTL SNP and each gene in the network, the user can prioritize genes with the most significant p values as likely *trans*-targets. Furthermore, the users can also use the $\pi1$ statistics on the univariate p values to estimate the proportion of genes that have true *trans* effects in the network. Although the exact molecular mechanism requires further validation, the large number of *trans*-eQTLs identified by *trans*-PCO in our study opens up new opportunities to understand complex traits-associated loci and underlying mechanisms.

STAR★METHODS

Detailed methods are provided in the online version of this paper and include the following:

- KEY RESOURCES TABLE
- RESOURCE AVAILABILITY
  - Lead contact
  - Materials availability
  - Data and code availability
- METHOD DETAILS
  - *Trans*-PCO pipeline
  - Simulation
  - Genotype QC of DGN dataset
  - Summary-statistics-based *trans*-PCO
- QUANTIFICATION AND STATISTICAL ANALYSIS
  - Colocalization of *trans*-eQTLs and GWAS loci
  - Colocalization of *trans*-eQTLs and *cis*-e/sQTLs
  - Trait heritability enrichment in gene modules
  - Association of drug targets with disease-relevant gene sets regulated in *trans*

SUPPLEMENTAL INFORMATION

Supplemental information can be found online at https://doi.org/10.1016/j.xgen.2024.100538.

ACKNOWLEDGMENTS

We thank Y. Li, A. Dahl, Y. Gilad, and Z. Mu for helpful discussions. We thank N. Gonzales, C. Jones, and S. Sumner for editing the manuscript. This work was completed in part with resources provided by the University of Chicago's Research Computing Center. This research was funded by the NIGMS Maximizing Investigators' Research Award (R35GM138084).

AUTHOR CONTRIBUTIONS

L.W., Z.L., and X.L. developed the method. L.W. and X.L. designed all of the analyses. L.W. implemented the method and performed all of the data analyses under the supervision of X.L. L.W. and X.L. wrote the manuscript with input from all of the coauthors. N.B. created the website to share the results.

DECLARATION OF INTERESTS

The authors declare no competing interests.

Received: May 10, 2023
Revised: December 8, 2023
Accepted: March 13, 2024
Published: April 1, 2024

REFERENCES

1. Maurano, M.T., Humbert, R., Rynes, E., Thurman, R.E., Haugen, E., Wang, H., Reynolds, A.P., Sandstrom, R., Qu, H., Brody, J., et al. (2012). Systematic Localization of Common Disease-Associated Variation in Regulatory DNA. Science *337*, 1190–1195. https://doi.org/10.1126/science.1222794.
2. Lango Allen, H., Estrada, K., Lettre, G., Berndt, S.I., Weedon, M.N., Rivadeneira, F., Willer, C.J., Jackson, A.U., Vedantam, S., Raychaudhuri, S., et al. (2010). Hundreds of variants clustered in genomic loci and biological pathways affect human height. Nature *467*, 832–838. https://doi.org/10.1038/nature09410.
3. Hindorff, L.A., Sethupathy, P., Junkins, H.A., Ramos, E.M., Mehta, J.P., Collins, F.S., and Manolio, T.A. (2009). Potential etiologic and functional implications of genome-wide association loci for human diseases and traits. Proc. Natl. Acad. Sci. USA *106*, 9362–9367. https://doi.org/10.1073/pnas.0903103106.
4. Watanabe, K., Stringer, S., Frei, O., Umićević Mirkov, M., de Leeuw, C., Polderman, T.J.C., van der Sluis, S., Andreassen, O.A., Neale, B.M., and Posthuma, D. (2019). A global overview of pleiotropy and genetic architecture in complex traits. Nat. Genet. *51*, 1339–1348. https://doi.org/10.1038/s41588-019-0481-0.

CellPress
OPEN ACCESS

5. Nicolae, D.L., Gamazon, E., Zhang, W., Duan, S., Dolan, M.E., and Cox, N.J. (2010). Trait-associated SNPs are more likely to be eQTLs: annotation to enhance discovery from GWAS. PLoS Genet. *6*, e1000888.
6. Liu, X., Li, Y.I., and Pritchard, J.K. (2019). Trans Effects on Gene Expression Can Drive Omnigenic Inheritance. Cell *177*, 1022–1034.e6. https://doi.org/10.1016/j.cell.2019.04.014.
7. Saha, A., and Battle, A. (2018). False positives in trans-eQTL and co-expression analyses arising from RNA-sequencing alignment errors. F1000Res. *7*, 1860. https://doi.org/10.12688/f1000research.17145.2.
8. Albert, F.W., Bloom, J.S., Siegel, J., Day, L., and Kruglyak, L. (2018). Genetics of trans-regulatory variation in gene expression. Elife *7*, e35471. https://doi.org/10.7554/eLife.35471.
9. Võsa, U., Claringbould, A., Westra, H.-J., Bonder, M.J., Deelen, P., Zeng, B., Kirsten, H., Saha, A., Kreuzhuber, R., Yazar, S., et al. (2021). Large-scale cis- and trans-eQTL analyses identify thousands of genetic loci and polygenic scores that regulate blood gene expression. Nat. Genet. *53*, 1300–1310. https://doi.org/10.1038/s41588-021-00913-z.
10. Rotival, M., Zeller, T., Wild, P.S., Maouche, S., Szymczak, S., Schillert, A., Castagné, R., Deiseroth, A., Proust, C., Brocheton, J., et al. (2011). Integrating Genome-Wide Genetic Variations and Monocyte Expression Data Reveals Trans-Regulated Gene Modules in Humans. PLoS Genet. *7*, e1002367. https://doi.org/10.1371/journal.pgen.1002367.
11. Kolberg, L., Kerimov, N., Peterson, H., and Alasoo, K. (2020). Co-expression analysis reveals interpretable gene modules controlled by trans-acting genetic variants. Elife *9*, e58705. https://doi.org/10.7554/eLife.58705.
12. Dutta, D., He, Y., Saha, A., Arvanitis, M., Battle, A., and Chatterjee, N. (2022). Aggregative trans-eQTL analysis detects trait-specific target gene sets in whole blood. Nat. Commun. *13*, 4323. https://doi.org/10.1038/s41467-022-31845-9.
13. Hore, V., Viñuela, A., Buil, A., Knight, J., McCarthy, M.I., Small, K., and Marchini, J. (2016). Tensor decomposition for multiple-tissue gene expression experiments. Nat. Genet. *48*, 1094–1100. https://doi.org/10.1038/ng.3624.
14. Aschard, H., Vilhjálmsson, B.J., Greliche, N., Morange, P.-E., Trégouët, D.A., and Kraft, P. (2014). Maximizing the Power of Principal-Component Analysis of Correlated Phenotypes in Genome-wide Association Studies. Am. J. Hum. Genet. *94*, 662–676. https://doi.org/10.1016/j.ajhg.2014.03.016.
15. Liu, Z., and Lin, X. (2019). A Geometric Perspective on the Power of Principal Component Association Tests in Multiple Phenotype Studies. J. Am. Stat. Assoc. *114*, 975–990. https://doi.org/10.1080/01621459.2018.1513363.
16. Battle, A., Mostafavi, S., Zhu, X., Potash, J.B., Weissman, M.M., McCormick, C., Haudenschild, C.D., Beckman, K.B., Shi, J., Mei, R., et al. (2014). Characterizing the genetic basis of transcriptome diversity through RNA-sequencing of 922 individuals. Genome Res. *24*, 14–24. https://doi.org/10.1101/gr.155192.113.
17. Liu, X., Mefford, J.A., Dahl, A., He, Y., Subramaniam, M., Battle, A., Price, A.L., and Zaitlen, N. (2020). GBAT: a gene-based association test for robust detection of trans-gene regulation. Genome Biol. *21*, 211. https://doi.org/10.1186/s13059-020-02120-1.
18. Langfelder, P., and Horvath, S. (2008). WGCNA: an R package for weighted correlation network analysis. BMC Bioinf. *9*, 559. https://doi.org/10.1186/1471-2105-9-559.
19. Liberzon, A., Birger, C., Thorvaldsdóttir, H., Ghandi, M., Mesirov, J.P., and Tamayo, P. (2015). The Molecular Signatures Database Hallmark Gene Set Collection. Cell Syst. *1*, 417–425. https://doi.org/10.1016/j.cels.2015.12.004.
20. Kanehisa, M., Furumichi, M., Sato, Y., Ishiguro-Watanabe, M., and Tanabe, M. (2021). KEGG: integrating viruses and cellular organisms. Nucleic Acids Res. *49*, D545–D551. https://doi.org/10.1093/nar/gkaa970.
21. Szklarczyk, D., Gable, A.L., Nastou, K.C., Lyon, D., Kirsch, R., Pyysalo, S., Doncheva, N.T., Legeay, M., Fang, T., Bork, P., et al. (2021). The STRING database in 2021: customizable protein–protein networks, and functional characterization of user-uploaded gene/measurement sets. Nucleic Acids Res. *49*, D605–D612. https://doi.org/10.1093/nar/gkaa1074.
22. Kim, S., and Xing, E.P. (2009). Statistical Estimation of Correlated Genome Associations to a Quantitative Trait Network. PLoS Genet. *5*, e1000587. https://doi.org/10.1371/journal.pgen.1000587.
23. Mu, Z., Wei, W., Fair, B., Miao, J., Zhu, P., and Li, Y.I. (2021). The impact of cell type and context-dependent regulatory variants on human immune traits. Genome Biol. *22*, 122. https://doi.org/10.1186/s13059-021-02334-x.
24. Giambartolomei, C., Vukcevic, D., Schadt, E.E., Franke, L., Hingorani, A.D., Wallace, C., and Plagnol, V. (2014). Bayesian Test for Colocalisation between Pairs of Genetic Association Studies Using Summary Statistics. PLoS Genet. *10*, e1004383. https://doi.org/10.1371/journal.pgen.1004383.
25. Westra, H.-J., Peters, M.J., Esko, T., Yaghootkar, H., Schurmann, C., Kettunen, J., Christiansen, M.W., Fairfax, B.P., Schramm, K., Powell, J.E., et al. (2013). Systematic identification of trans eQTLs as putative drivers of known disease associations. Nat. Genet. *45*, 1238–1243. https://doi.org/10.1038/ng.2756.
26. Luijk, R., Dekkers, K.F., van Iterson, M., Arindrarto, W., Claringbould, A., Hop, P., Boomsma, D.I., van Duijn, C.M., van Greevenbroek, M.M.J., Veldink, J.H., et al. (2018). Genome-wide identification of directed gene networks using large-scale population genomics data. Nat. Commun. *9*, 3097. https://doi.org/10.1038/s41467-018-05452-6.
27. Morris, J.A., Caragine, C., Daniloski, Z., Domingo, J., Barry, T., Lu, L., Davis, K., Ziosi, M., Glinos, D.A., Hao, S., et al. (2023). Discovery of target genes and pathways at GWAS loci by pooled single-cell CRISPR screens. Science *380*, eadh7699. https://doi.org/10.1126/science.adh7699.
28. Raudvere, U., Kolberg, L., Kuzmin, I., Arak, T., Adler, P., Peterson, H., and Vilo, J. (2019). g:Profiler: a web server for functional enrichment analysis and conversions of gene lists (2019 update). Nucleic Acids Res. *47*, W191–W198. https://doi.org/10.1093/nar/gkz369.
29. Schwickert, T.A., Tagoh, H., Gültekin, S., Dakic, A., Axelsson, E., Minnich, M., Ebert, A., Werner, B., Roth, M., Cimmino, L., et al. (2014). Stage-specific control of early B cell development by the transcription factor Ikaros. Nat. Immunol. *15*, 283–293. https://doi.org/10.1038/ni.2828.
30. Lemarié, M., Bottardi, S., Mavoungou, L., Pak, H., and Milot, E. (2021). IKAROS is required for the measured response of NOTCH target genes upon external NOTCH signaling. PLoS Genet. *17*, e1009478. https://doi.org/10.1371/journal.pgen.1009478.
31. Cui, J., Zhu, L., Xia, X., Wang, H.Y., Legras, X., Hong, J., Ji, J., Shen, P., Zheng, S., Chen, Z.J., and Wang, R.F. (2010). NLRC5 Negatively Regulates the NF-κB and Type I Interferon Signaling Pathways. Cell *141*, 483–496. https://doi.org/10.1016/j.cell.2010.03.040.
32. Kobayashi, K.S., and van den Elsen, P.J. (2012). NLRC5: a key regulator of MHC class I-dependent immune responses. Nat. Rev. Immunol. *12*, 813–820. https://doi.org/10.1038/nri3339.
33. Bycroft, C., Freeman, C., Petkova, D., Band, G., Elliott, L.T., Sharp, K., Motyer, A., Vukcevic, D., Delaneau, O., O'Connell, J., et al. (2018). The UK Biobank resource with deep phenotyping and genomic data. Nature *562*, 203–209. https://doi.org/10.1038/s41586-018-0579-z.
34. Liu, J.Z., van Sommeren, S., Huang, H., Ng, S.C., Alberts, R., Takahashi, A., Ripke, S., Lee, J.C., Jostins, L., Shah, T., et al. (2015). Association analyses identify 38 susceptibility loci for inflammatory bowel disease and highlight shared genetic risk across populations. Nat. Genet. *47*, 979–986. https://doi.org/10.1038/ng.3359.
35. De Lange, K.M., Moutsianas, L., Lee, J.C., Lamb, C.A., Luo, Y., Kennedy, N.A., Jostins, L., Rice, D.L., Gutierrez-Achury, J., Ji, S.G., et al. (2017). Genome-wide association study implicates immune activation of multiple integrin genes in inflammatory bowel disease. Nat. Genet. *49*, 256–261. https://doi.org/10.1038/ng.3760.

36. Ferreira, M.A., Vonk, J.M., Baurecht, H., Marenholz, I., Tian, C., Hoffman, J.D., Helmer, Q., Tillander, A., Ullemar, V., van Dongen, J., et al. (2017). Shared genetic origin of asthma, hay fever and eczema elucidates allergic disease biology. Nat. Genet. *49*, 1752–1757. https://doi.org/10.1038/ng.3985.

37. Loh, P.-R., Kichaev, G., Gazal, S., Schoech, A.P., and Price, A.L. (2018). Mixed-model association for biobank-scale datasets. Nat. Genet. *50*, 906–908. https://doi.org/10.1038/s41588-018-0144-6.

38. International Multiple Sclerosis Genetics Consortium (2019). Multiple sclerosis genomic map implicates peripheral immune cells and microglia in susceptibility. Science *365*, eaav7188. https://doi.org/10.1126/science.aav7188.

39. Ferreira, M.A.R., Mathur, R., Vonk, J.M., Szwajda, A., Brumpton, B., Granell, R., Brew, B.K., Ullemar, V., Lu, Y., Jiang, Y., et al. (2019). Genetic Architectures of Childhood- and Adult-Onset Asthma Are Partly Distinct. Am. J. Hum. Genet. *104*, 665–684. https://doi.org/10.1016/j.ajhg.2019.02.022.

40. Bentham, J., Morris, D.L., Graham, D.S.C., Pinder, C.L., Tombleson, P., Behrens, T.W., Martín, J., Fairfax, B.P., Knight, J.C., Chen, L., et al. (2015). Genetic association analyses implicate aberrant regulation of innate and adaptive immunity genes in the pathogenesis of systemic lupus erythematosus. Nat. Genet. *47*, 1457–1464. https://doi.org/10.1038/ng.3434.

41. Zou, S., Teixeira, A.M., Kostadima, M., Astle, W.J., Radhakrishnan, A., Simon, L.M., Truman, L., Fang, J.S., Hwa, J., Zhang, P.X., et al. (2017). SNP in human ARHGEF3 promoter is associated with DNase hypersensitivity, transcript level and platelet function, and Arhgef3 KO mice have increased mean platelet volume. PLoS One *12*, e0178095. https://doi.org/10.1371/journal.pone.0178095.

42. Finucane, H.K., Bulik-Sullivan, B., Gusev, A., Trynka, G., Reshef, Y., Loh, P.-R., Anttila, V., Xu, H., Zang, C., Farh, K., et al. (2015). Partitioning heritability by functional annotation using genome-wide association summary statistics. Nat. Genet. *47*, 1228–1235. https://doi.org/10.1038/ng.3404.

43. Rowland, B., Venkatesh, S., Tardaguila, M., Wen, J., Rosen, J.D., Tapia, A.L., Sun, Q., Graff, M., Vuckovic, D., Lettre, G., et al. (2022). Transcriptome-wide association study in UK Biobank Europeans identifies associations with blood cell traits. Hum. Mol. Genet. *31*, 2333–2347. https://doi.org/10.1093/hmg/ddac011.

44. Artavanis-Tsakonas, S., Rand, M.D., and Lake, R.J. (1999). Notch Signaling: Cell Fate Control and Signal Integration in Development. Science *284*, 770–776. https://doi.org/10.1126/science.284.5415.770.

45. Taylor-Weiner, A., Aguet, F., Haradhvala, N.J., Gosai, S., Anand, S., Kim, J., Ardlie, K., Van Allen, E.M., and Getz, G. (2019). Scaling computational genomics to millions of individuals with GPUs. Genome Biol. *20*, 228. https://doi.org/10.1186/s13059-019-1836-7.

46. GTEx Consortium (2020). The GTEx Consortium atlas of genetic regulatory effects across human tissues. Science *369*, 1318–1330. https://doi.org/10.1126/SCIENCE.AAZ1776.

47. Liu, B., Gloudemans, M.J., Rao, A.S., Ingelsson, E., and Montgomery, S.B. (2019). Abundant associations with gene expression complicate GWAS follow-up. Nat. Genet. *51*, 768–769. https://doi.org/10.1038/s41588-019-0404-0.

CellPress
OPEN ACCESS

STAR★METHODS

KEY RESOURCES TABLE

REAGENT or RESOURCE	SOURCE	IDENTIFIER
Deposited data		
Data generated in this paper, including all *trans*-eQTL signals, with functional annotation of the gene sets	This paper	http://www.networks-liulab.org/transPCO Zenodo link: https://zenodo.org/doi/10.5281/zenodo.10602699
Depression Genes and Networks study (DGN)	Battle et al.[16]	Downloaded by application through the NIMH Center for Collaborative Genomic Studies on Mental Disorders, under the "Depression Genes and Networks study (D. Levinson, PI)."
eQTLGen summary statistics	Võsa et al.[9]	https://www.eqtlgen.org/
MSigDB hallmark gene sets	Liberzon et al.[19]	http://www.gsea-msigdb.org/gsea/msigdb/human/genesets.jsp?collection=H
UK Biobank GWAS summary statistics	http://www.nealelab.is/uk-biobank/	http://www.nealelab.is/uk-biobank/
The ENCODE 36-mer of the reference human genome	N/A	https://hgdownload.soe.ucsc.edu/goldenPath/hg19/encodeDCC/wgEncodeMapability/wgEncodeCrgMapabilityAlign36mer.bigWig
Software and algorithms		
All original code, related to the *trans*-PCO pipeline and code to reproduce analyses presented in this work	This paper	https://github.com/liliw-w/Trans Zenodo link: https://zenodo.org/doi/10.5281/zenodo.10602558
TensorQTL	Taylor-Weiner et al.[45]	https://github.com/broadinstitute/tensorqtl
WGCNA	Langfelder et al.[18]	https://cran.r-project.org/web/packages/WGCNA/index.html
coloc	Giambartolomei et al.[24]	https://github.com/chr1swallace/coloc
S-LDSC	Finucane et al.[42]	https://github.com/bulik/ldsc

RESOURCE AVAILABILITY

Lead contact

Further information and requests for resources and reagents should be directed to and will be fulfilled by the lead contact, Xuanyao Liu (xuanyao@uchicago.edu).

Materials availability

No materials were generated in the study.

Data and code availability

- All *trans*-eQTL signals, with functional annotation of the gene sets, can be browsed and downloaded at http://www.networks-liulab.org/transPCO, and has been deposited at Zenodo (https://zenodo.org/doi/10.5281/zenodo.10602699). Any additional data reported in this paper will be shared by the lead contact upon request. DOIs are listed in the key resources table.
- All original code, related to the *trans*-PCO pipeline and code to reproduce analyses presented in this work are publicly available at https://github.com/liliw-w/Trans, and has been deposited at Zenodo (https://zenodo.org/doi/10.5281/zenodo.10602558). DOIs are listed in the key resources table.
- Any additional information required to reanalyze the data reported in this paper is available from the lead contact upon request.

METHOD DETAILS

Trans-PCO pipeline

Data processing

Trans-PCO removes all reads that are mapped to low mappability regions, in addition to multi-mapped reads marked by alignment tools before quantifying gene expression levels. More specifically, we downloaded the mappability of 36-mer of the reference human

genome computed by the ENCODE project and defined genomic regions with a mappability score <1 (i.e., 36-mers that could be mapped to two or more different genomic regions) as low mappability regions. We removed reads mapped to low mappability regions allowing 2 mismatches.

Following thorough read removal, *trans*-PCO quantifies gene expression levels as Transcript Per Million (TPM). Gene expression levels were first quantile normalized across samples, and then normalized to a standard normal across genes. We also filtered out genes that are not protein-coding or lincRNA genes. Finally, to control for potential confounding factors and capture the co-expressed gene modules mainly driven by genetic effects, we regressed out covariates from the expression profiles. The typical covariates may include biological and technical covariates, such as genotype PCs, expression PCs, and blood cell type proportions etc.[16,17]

Identification of gene co-expression networks

By default, *trans*-PCO uses WGCNA[18] to construct gene co-expression modules, where genes are connected through correlations among their residualized expression levels. WGCNA uses hierarchical clustering to cut the network into separate gene modules with highly correlated expression levels. We used the default parameter settings, except that we specified the minimum module size parameter ('minModuleSize') to 10 to obtain small gene modules. *Trans*-PCO also takes other pre-defined gene sets, such as genes in the same pathway or biological processes.

Multivariate association test

We test if a genetic variant is associated with genes in a module through *trans* regulations using the multivariate model as follows,

$$[y_1 \cdots y_K] = G[\beta_1 \cdots \beta_K] + covariates + e$$

where G is the dosage of a reference allele representing the genotype of an SNP, β_k is the effect of the SNP on k-th gene in the module with K genes, and y_k is the expression level of the k-th gene. To test if an SNP of interest is significantly associated with the module, we test the null hypothesis,

$$H_0 : \beta_1 = \cdots = \beta_K = 0$$

We use a PC-based omnibus test (PCO),[15] which is a powerful and robust PC-based approach aiming at testing genetic association with multiple genes with no prior knowledge of the true effects.

Specifically, PCO combines multiple single PC-based tests in linear and non-linear ways, corresponding to a range of causal relationships between the genetic variant and genes, to achieve higher power and better robustness. A single PC-based test (most commonly the first primary PC_1) is,

$$T_{PC_k} = {\mu_k}^T Z \sim N\left({\mu_k}^T \beta, \lambda_k\right), 1 \leq k \leq K$$

where Z is a $K \times 1$ vector of univariate summary statistic Z scores of the SNP for K genes in a module, μ_k is the k-th eigenvector of the covariance matrix $\Sigma_{K\times K}$ of Z, λ_k is the corresponding eigenvalue, and β represents the true causal effect. PCO combines six PC-based tests, including,

$$PCMinP = min_{1 \leq k \leq K} p_k, \text{and } PCFisher = -2\sum_{k=1}^{K} log(p_k),$$

where p_k is the p value of T_{PC_k}. These two tests take the best p value of single PC-based tests and combine multiple PC p values as the test statistic. Other tests include,

$$PCLC = \sum_{k=1}^{K} \frac{T_{PC_k}}{\lambda_k}, WI = \sum_{k=1}^{K} {T_{PC_k}}^2, Wald = \sum_{k=1}^{K} \frac{{T_{PC_k}}^2}{\lambda_k}, VC = \sum_{k=1}^{K} \frac{{T_{PC_k}}^2}{\lambda_k^2}$$

which are linear and quadratic combinations of each single PC-based test weighted by eigenvalues. The six tests achieve best power in specific genetic settings with different true causal effects.[15] PCO takes the best p value of the PC-based tests as the final test statistic,

$$T_{PCO} = min\ p_{\{PCMinP, PCFisher, PCLC, WI, Wald, VC\}}$$

to achieve robustness under unknown genetic architectures while maintaining a high power. The p value of PCO test statistics can be computed by performing an inverse-normal transformation of the test statistics,

$$p_{T_{PCO}} = 1 - P\left\{min\ \Phi^{-1}\left(p_{\{PCMinP, PCFisher, PCLC, WI, Wald, VC\}}\right) > \Phi^{-1}\left(T_{PCO}^{obs}\right)\right\}$$

where Φ^{-1} denotes the inverse standard normal cumulative distribution function. The p value can be efficiently computed using a multivariate normal distribution as described in Liu et al.[15]

To prevent *cis*-regulatory effects from driving the identified *trans* associations between an SNP and module, we removed genes in the module that are on the same chromosome as the tested variant. In addition, to avoid false positive signals in *trans* associations

due to alignment errors, we discarded RNA-seq reads that are mapped to multiple locations or poorly mapped genomic regions (mappability score <1)[16,17] before quantifying gene expression levels. We calculated the summary statistic Z scores using TensorQTL.[45]

Simulation

We performed simulations to evaluate power and type I error of *trans*-PCO, a univariate test ("MinP") and a primary PC-based test ("PC1"). The PC1 based test takes only the first PC as the proxy of a gene module and uses it as the response variable to test for genetic variants with significant associations. The MinP method uses the minimum p value across genes in the module to represent the association of the gene module. More specifically, the test statistics of the PC1 method is $T_{PC_1} = {\mu_1}^T Z \sim N({\mu_1}^T \beta, \lambda_1)$, where Z is the vector of z scores between the SNPs and each individual gene, μ_1 is the first eigenvector of the covariance matrix Σ_K of the K genes, λ_1 is the corresponding eigenvalue, and β represents the true causal effect. The p value of PC1 test statistics is computed based on $N({\mu_1}^T \beta, \lambda_1)$. The test statistics of the MinP method is $T_{MinP} = min\{p_1^g, \cdots, p_K^g\}$, where p_i^g is the association p value of gene i. The p value of MinP test statistics is $P_{MinP} = T_{MinP} \times K$, which uses Bonferroni correction.

We used a real gene module containing 101 genes (Module 29) from the DGN dataset in our simulations. The correlation matrix of the 101 genes is Σ_{101}. In null simulations, we simulated Z scores of 10^7 SNPs from the null distribution, $Z_{NULL} \sim N(0, \Sigma_{101})$. We applied the three methods to the simulated Z scores and evaluated the p values to validate if the statistical tests are well calibrated for type I error.

In power simulations, we simulated 10k Z scores of SNPs from the alternative distribution,

$$Z_{Alt} \sim N\left(\sqrt{n}\left[\beta_{101\gamma}, 0\right]^T, \Sigma_{101}\right)$$

where n is the sample size, β is a 101γ-long vector representing the causal effect of an SNP on 101 genes, and γ is the proportion of true target genes in the module with non-zero effects. We generated β_k from a point normal distribution, where $\beta_k \sim N(0, \sigma_b^2)$ for proportion γ, and $\beta_k = 0$, otherwise. The *trans*-genetic variance is σ_b^2, which is a low and realistic per SNP heritability for *trans* effects. By default, we set the sample size n to be 500, 30% genes (30) in the module are true *trans* target genes, and σ_b^2 to be 0.001.

To evaluate how three tests perform across different genetic architectures, we simulated multiple scenarios across varying sample sizes, target gene proportions, and genetic variances. Specifically, we looked at the cases where sample size is 200, 400, 600, and 800, causal genes proportion is 1%, 5%, 10%, 30%, and 50%, and genetic variance is 0.002, 0.003, 0.004, 0.005, and 0.006. We simulated 10k SNPs and performed 1000 simulations. To control the false discovery rate, we corrected the p values for multiple testing based on the simulated empirical null distribution of p values, to keep it consistent with the method used in the RNA-seq dataset (Methods S1). We set significance levels at 10% FDR to be consistent with real data analysis. We computed power as the average proportion of significant tests out of 10,000 simulated SNPs across 1000 simulations. An association is significant if its adjusted p value is lower than 0.1. We computed power as the average proportion of significant tests out of 10,000 simulated SNPs across 1000 simulations.

Genotype QC of DGN dataset

We analyzed an RNA-seq dataset from whole blood.[16] We performed a series of QC on individuals, genotypes, RNA-seq reads, and genes before quantifying gene expression profiles. The QC of RNA-seq data and quantifying gene expression is included in the preprocessing steps of *trans*-PCO (see above). For individual-level QC, we removed related individuals from 922 samples and kept 913 individuals in total for further analysis. For genotype-level QC, we used SNPs with genotyping rate >99%, minor allele frequency >5%, and Hardy-Weinberg equilibrium < 10^{-6}. The detailed procedures were described in Liu et al.[17]

Summary-statistics-based *trans*-PCO

The eQTLGen Consortium[9] has conducted the largest *cis*- and *trans*-eQTLs association analyses in blood to date. Specifically, 31,684 samples were tested for over 11 million SNPs across 37 cohorts. The summary statistics of *trans*-eQTLs are available for 10,317 trait-associated SNPs on 19,942 genes.

We applied our pipeline *trans*-PCO to eQTLGen summary statistics, using the same 166 co-expression gene modules defined in DGN dataset. We searched for *trans*-eQTLs among 10,317 SNPs.

The eQTLGen summary statistics are marginal Z scores meta-weighted across multiple cohorts. Most Z scores are from studies where the RNA-seq reads with mappability issues were not filtered out before quantifying gene expression profiles. Therefore, directly applying *trans*-PCO to the summary statistics can lead to false positive signals, which are driven by the cross-mappability between the genes in the module and the *cis*-gene of the test SNP. In order to reduce false positive *trans* signals, we removed from the gene module genes that are cross-mappable to the *cis*-gene (within 100kb) of the test SNP, which is a common practice used in previous studies.[7,16,46] We further removed genes on the same chromosome as the test SNP to prevent the detected *trans* effects from being dominated by *cis* regulations.

The gene expression profiles are not available in eQTLGen. Therefore, to estimate the gene correlation Σ of a module, we searched among eQTLGen SNPs for SNPs insignificantly associated with the module (null SNPs) (see Methods S3 for details, Figure S20). We observed that there are less null SNPs that can be found for large modules. And simulations show that the low ratio of the number of

null SNPs used for Σ estimation to the module size leads to false positive signals (Methods S3). Therefore, we removed 37 gene modules with ratios lower than 50. Finally, we performed *trans*-PCO on the remaining 129 gene modules.

QUANTIFICATION AND STATISTICAL ANALYSIS

Colocalization of *trans*-eQTLs and GWAS loci

To define a region to perform colocalization, we first selected the *trans*-eQTL with the most significant p value and expanded a 200kb flanking genomic region centered at the lead SNP as a region to perform colocalization analysis. We then moved on to the next most significant SNP and expanded a 200kb flanking region. We stopped searching for lead SNPs when all *trans*-eQTLs were included. This resulted in 255 *trans* region-module pairs. As two adjacent regions could correspond to the same colocalization signal, we marked adjacent regions as a region group if their lead SNPs were within 200kb, which generated 179 *trans*-region–module pairs in total. We ran colocalization analysis between each 200kb *trans* region and GWAS loci of 46 complex traits using the R package coloc,[24] assuming there is at most one causal variant for each region. We used the default priors and 0.75 as the PP4 cutoff for significant colocalizations. We defined a merged region group as being colocalized with a trait if any of its 200kb sub-regions has significant colocalization with the trait. We visualized the colocalized regions using LocusCompareR.[47]

Colocalization of *trans*-eQTLs and *cis*-e/sQTLs

We performed colocalization analysis between *trans*-eQTLs and *cis*-eQTLs (*cis*-sQTLs) of genes near the *trans*-eQTLs. We used the same 179 *trans*-region–module pairs defined in the colocalization analysis of GWAS loci. For a *trans* loci, we searched for the genes within 500 kb around the lead *trans*-eQTLs of the loci, and used these genes to perform colocalization. We used summary statistics of *cis*-eQTLs and *cis*-sQTLs in the DGN dataset from Mu et al.[23] We ran coloc[24] with default priors and 0.75 as PP4 cutoff.

Trait heritability enrichment in gene modules

To investigate whether a gene module is enriched for trait heritability, we applied stratified LD score regression[42] (S-LDSC) to 166 co-expression gene modules and 46 complex traits and diseases. Specifically, for each module we defined the annotation set as the SNPs within genomic regions of genes in the module and also a 500 base-pair window around the genes. We also included 97 annotations from the baseline model. Partitioned heritability enrichment was calculated as the proportion of trait heritability contributed by SNPs in the module annotation over the proportion of SNPs in that annotation.

Association of drug targets with disease-relevant gene sets regulated in *trans*

To show the translational application of *trans*-PCO results, we examined whether drug targets are more likely to be associated with disease-relevant pathways or gene sets in *trans* (Methods S4). We first downloaded drug targets of various diseases from The Broad Institute Drug Repurposing Hub (https://repo-hub.broadinstitute.org/repurposing). We then examined whether the drug targets are near any SNPs that have significant trans associations with immune-related gene co-expression modules or hallmark gene sets in the eQTLGen dataset.

Cell Genomics, Volume *4*

Supplemental information

Trans-eQTL mapping in gene sets identifies network effects of genetic variants

Lili Wang, Nikita Babushkin, Zhonghua Liu, and Xuanyao Liu

Supplemental Methods

Method S1. Trans-PCO on individual level RNA-seq data (Related to STAR Methods)

Estimating correlation matrix

To implement trans-PCO on a pair of SNP and gene module, we need two pieces of information, (i) z-score vector of the SNP on each individual genes in the module, and (ii) estimated Σ of the module. In the analysis of DGN dataset[1], we calculated the summary statistic z-scores using TensorQTL[2] as described in Method details.

In the analysis of DGN dataset[1], we calculated the summary statistic z-scores using TensorQTL[2] as described in Method details.which performs ultrafast *trans*-QTL mapping. We included 74 biological and technical factors as covariates, including 5 genotype PCs, 10 expression PCs, and the estimated blood cell type proportions etc[1,3]. In order to search along the whole genome for trans-eQTLs of gene modules, we calculated genome-wide z-scores for every gene included in all modules (over 10k genes).

There are two ways to estimate Σ of a gene module[4]. The first way is to use correlation matrix of the residualized gene expression levels,

$$\Sigma = cor(Y|covariates),$$

where $Y|covariates$ is the residual gene expression levels after regressing out covariates. We used this estimation when gene expression profiles are available, as in the case of DGN.

The other way to estimate Σ is to use the covariance matrix of insignificant z-scores (null z-scores) across genes in the module[4],

$$\Sigma = cor(Z),$$

where Z is the z-score matrix of null independent SNPs and genes in the module. We used this estimation when only summary statistics are available, as in the analysis of eQTLGen. More specifically, we collected a large set of independent null SNPs, took the z-scores of SNPs across module genes, and calculated the sample covariance matrix using z-scores over the independent null SNPs. See more details in “Summary statistics based trans-PCO”.

Multiple testing correction

In the case of analyzing DGN dataset, we tested the genome-wide associations of 166 co-expression gene modules. To correct for multiple testing, we used the empirical null distribution. Specifically, we first randomly permuted sample labels and obtained the null summary statistics across SNPs and genes. Then, we calculated the associations for each pair of SNPs and modules using the null z-scores, and used the p-values as the empirical null

distribution. Finally, we corrected each observed p-value by counting how many null tests fall below the observed p-value. We did ten permutations and used the average corrected p-values to claim significance (FDR<0.1). By using the empirical null distribution to correct p-values, we are able to control the false positive rate and preserve the LD structure among SNPs as well as the correlation structure within modules.

PCs included in trans-PCO

Trans-PCO tests multiple genes jointly by combining multiple PC's of the genes. However, it is not always best to use all PC's, as suggested by Liu et al.[4], due to the tradeoff between statistical power and numerical stability. Particularly, for a large module with highly correlated genes, the correlation matrix Σ would be close to being ill-conditioned. Therefore, the eigenvalues of the last few PC's would be very small, which can lead to inflated test statistics. For example, VC test (one of the six tests PCO constructs its test statistics on),

$$VC = \sum_{k=1}^{K} \frac{T_{PC_k}^{\ 2}}{\lambda_k^2},$$

is weighted by the inverse of eigenvalues, where K is the module size, λ_k is k-th eigenvalue, and T_{PC_k} is the k-th PC test statistic. The VC test can be numerically unstable if it includes the last few PC's that have very small eigenvalues.

As a matter fact, we observed a few modules with very small eigenvalues (Figure S1). To investigate how including PC's with small eigenvalues affects the association tests, we did simulations where we performed tests incorporating different numbers of PC's. We found that by including PC's with extremely small eigenvalues, the p-values of null tests were inflated. Therefore, instead of combining all PC's, we discarded the last few PC's with extremely small eigenvalues and used only the top PC's with eigenvalues larger than 0.01.

Method S2. Compare trans-PCO with existing methods (Related to STAR Methods, Figure 2, and Figure 3)

We compared trans-PCO to a few other methods, including the primary PC method proposed by Kolberg et al.[7], ARCHIE method proposed by Dutta et al.[8], and a method proposed by Rotival et al.[10]. We provided thorough comparisons to the three methods in simulations and/or real data analyses.

Compare trans-PCO and primary PC method

The Kolberg et al. method[7] applies the similar idea of first constructing co-expression modules and then testing associations between a variant and a group of genes. The method essentially uses the first primary PC (PC1) of a gene module to represent the co-expression pattern among genes. Specifically, the method first infers gene co-expression modules using two types of methods - co-expression clustering (WGCNA and funcExplorer) or matrix factorisation (PLIER, ICA and PEER) methods. Each gene co-expression module is represented by a single

‘eigengene’ that captures the co-expression correlation within the module. As noted in Kolberg et al.[7], the ‘eigengene’ is essentially PC1 of a gene module or highly correlated with PC1.

Apply the primary PC method to DGN dataset

To compare the primary PC method[7] that includes only the first PC in the test and trans-PCO that combines multiple PC’s, we also applied the primary PC approach to detect *trans*-eQTLs of the RNA-seq dataset (DGN). Specifically, the test statistic we used to test a pair of SNP and gene module is,

$$T_{PC_1} = \mu_1^T Z,$$

where Z is the z-score vector of the SNP over the gene module, μ_1 is the eigenvector of the first PC. The idea is to utilize the first PC of the module as a one-dimensional proxy phenotype to represent the module and then test its association with SNPs.

The procedure of applying the primary PC method to DGN is similar to that of applying trans-PCO. Specifically, we started with the processed gene expression profiles by removing RNA-seq reads that were poorly mapped or cross-mapped to multiple genomic regions (see Methods). We then regressed out the biological and technical covariates as in trans-PCO. We obtained the first PC from the residual gene expression levels to calculate the test statistic. We also constructed the co-expression gene network from the residualized expression levels, and tested the same set of 166 co-expression gene modules defined as in DGN dataset. We also removed genes from the tested module that are located on the same chromosome as the tested SNP, to avoid the confusion of *cis* effects. We then performed a genome-wide scan of *trans*-eQTLs of 166 modules using the primary PC method. We corrected for multiple testing based on the empirical null distribution of p-values obtained by permuting sample labels. We did ten permutations. An association is claimed to be significant if the average corrected p-value is under 0.1.

Performance comparison in simulations and real dataset

Previous studies have shown that PC1 has very limited power in identifying genetic effects on multiple genes, even though PC1 captures the largest amount of total variance in gene expression levels. Higher order PCs may have better power than PC1, but it is hard to predict which PCs to use to achieve the best power, as achieving high powers depends on not only a good representative of a gene module, but also the true genetic effects of variants on genes in the module, which is unknown in practice. Therefore, to achieve high and robust power, we used the omnibus PC-based test (PCO), which uses multiple PCs and combines these PCs in several linear and nonlinear ways to capture various genetic effects under different genetic architectures[4].

We performed a thorough comparison of our method and Kolberg et al. both in simulation (Figure 2, Figure S2, Figure S9, Figure S8, Methods ‘Simulation’) and real data analysis (Figure S7, Supplementary Note ‘Apply the primary PC method to DGN dataset’). In simulations, we showed that the PC1 approach (i.e. Kolberg et al) indeed had limited power for detecting

trans-eQTLs associated with multiple genes' expression levels. For example, in Figure 2, the PC1 approach had a power of 0.0018% versus 74% of trans-PCO at the sample size of 800 under a specific but realistic simulation setting. In Figure S2, we showed that PC1 also had very limited power (near 0% power) in comparison to trans-PCO and the univariate approach under various simulation settings of genetic variance. We also applied the Kolberg et al. method to the real dataset DGN. In summary, Kolberg et al. method identified a much less number of *trans* signals than trans-PCO. More specifically, it identified 1483 significant *trans*-eSNP–module pairs (55 *trans*-loci–module pairs) at 10% FDR, and 1464 pairs (99%) were detected by trans-PCO (Figure S7). Overall, Kolberg et al. method identified 38% of *trans* signals detected by trans-PCO (Figure S7).

While this result supports that trans-PCO is more powerful than Kolberg et al. method, there were more signals detected by Kolberg et al. method in real data than expected from simulation results, where it remains powerless across almost all simulation settings. To address the discrepancy between the Kolberg et al. method performance in simulations versus real data, we performed additional simulations and analyses with real data (Figure S7, Figure S9, Figure S8).

We note that we simulated weak *trans* effects and sparse causal proportions in the simulations in order to better reflect common and realistic *trans* effects. To further represent general use cases, we did not specify the direction of the genetic effects on genes in a module in relation to the primary PC of the module. Although Kolberg et al. method remains powerless in these general simulation settings, we showed in additional simulations that it can have good statistical power in some specific cases. For example, statistically, PC1 can also have good statistical power when the *trans* effects align with the direction of PC1. To demonstrate this, we performed simulations under the assumption that the genetic effect vector perfectly aligns with the primary PC direction. We found that Kolberg et al. method has higher power than trans-PCO and MinP methods under this special circumstance (Figure S9). However, it is impossible to predict whether the directions would align in real data as the true genetic effects are unknown.

Additionally, we found that the Kolberg et al. method also gains power when the genetic effects are substantially large and the proportion of causal genes in the gene module is high. We performed simulations, where the genetic variance is 0.2 and the proportion of causal genes is 100%, the method has 50% power of identifying signals (Figure S8). Therefore, we expect some *trans* signals that are detected by the Kolberg et al. method in real data are likely to be among the strongest *trans* effects. We compared the univariate z-scores of *trans*-eQTLs detected by trans-PCO and the Kolberg et al. method (Figure S7B-D). We found that signals identified by the Kolberg et al. method have higher z-scores than signals detected by trans-PCO, supporting that the Kolberg et al. method detected those strong *trans* effects in real data, whereas trans-PCO detected additional *trans* signals with much weaker effects.

We did not apply the Kolberg et al. method to eQTLGen dataset, because their method is not applicable to summary-statistics level data. In contrast, trans-PCO can be applied to both raw individual level expression/genotype dataset and summary-statistical level data.

Through simulations and real data analyses, we conclude that trans-PCO is much more powerful than Kolberg et al. at identifying *trans*-eQTLs of gene sets. In addition, trans-PCO has wider applications as it does not require individual level data of gene expression and genotypes.

Compare trans-PCO and ARCHIE

We compared trans-PCO to the ARCHIE method proposed in Dutta et al.[8]. ARCHIE is a summary statistic-based method with the goal of identifying sets of gene expressions *trans-regulated* by sets of known trait-related SNPs. The key output of ARCHIE is ARCHIE components, which are a set of selected genes and a set of trait-relevant SNPs whose linear combinations have a high canonical correlation (cc-value) due to trait-specific *trans* regulations. Specifically, Dutta et al.[8] analyzed genetic variants associated with 29 traits using eQTLGen summary statistics. The authors made ARCHIE components of three traits (Figure S4) publicly available, including prostate cancer, schizophrenia, and ulcerative colitis.

ARCHIE and trans-PCO are designed with different goals and usages. First, *trans* regulations captured by ARCHIE components reflect only trait-specific associations. ARCHIE uses only variants specific to a specific trait as input and finds *trans* regulations by these variants. Additionally, ARCHIE tests significance against a competitive null hypothesis, which characterizes all trait-associated variants and reflects general *trans* regulations not specific to any trait, as trait-associated variants are expected to be enriched for *trans*-eQTLs[8]. Therefore, a p-value under the competitive null hypothesis reflects the significance of trait-specific patterns. In contrast, trans-PCO identifies all *trans*-eQTLs of given tissues and cell types under the general null hypothesis assuming no *trans* effects. In addition, using ARCHIE to perform genome-wide scan of *trans*-eQTLs in a non-trait specific manner can be challenging, as non-trait specific p-value is not computable in current implementation of the method and it will be extremely computational challenging (due to the computational intensive resampling procedure and difficulty of manipulating whole-genome LD matrices).

Second, trans-PCO and ARCHIE are designed to capture different *trans-regulatory* effects (Figure S4A). As shown in ARCHIE simulations[8], it is powerful in the case where one gene has multiple weak *trans* effects or more complicated *trans* effects between multiple SNPs and multiple genes. For example, multiple GWAS variants converge onto the core genes through *trans* regulation) or multiple disease–associated variants have weak effects on multiple genes (Figure 2 in Dutta et al.). In fact, they observed that a majority of novel genes they found have multiple weak *trans* associations with variants selected in ARCHIE components. In contrast, trans-PCO is powerful in detecting *trans* signals where a SNP has weak *trans* effects on multiple gene expressions as shown in our simulations, for example, a transcription factor has *trans* effects on multiple target genes.

Third, another difference lies in the way gene sets (modules) are defined by two methods for evaluating associations. Specifically, ARCHIE takes all genes as input and infers gene sets as gene components, whereas trans-PCO is flexible to be applied to any user-defined gene set (module) of interest, such as biological pathways or processes.

Lastly, ARCHIE identifies components, consisting of multiple trait-associated SNPs and multiple genes. The interpretation of an ARCHIE component is that sets of gene expressions are *trans* regulated by sets of trait-associated variants, without knowing which exact variant drives the association. ARCHIE components make it useful for identifying genes involved in traits through *trans* regulation, but not to identify *trans*-eQTL SNPs. In contrast, trans-PCO identifies associations between a single variant and multiple genes, which makes it easier to interpret *trans* signals (e.g., whether a *trans*-eQTL is a *cis*-eQTL or *cis*-sQTL, or whether the nearest gene to the top *trans*-eQTL is a transcription factor). Although ARCHIE can also be applied to single-variant cases, the power is extremely low (Figure S4E).

The differences in goals and usages of ARCHIE and trans-PCO make them not directly comparable. However, to give readers and users insights on when and how each method should be used, we compared their performance using three strategies: (1) in simulations, we evaluated how well ARCHIE can detect regular *trans*-eQTL signals between a single SNP and a gene set, and (2) in eQTLGen, we evaluated how well trans-PCO can replicate signals detected by ARCHIE, and (3) we compared *trans* signals reported by trans-PCO and ARCHIE in the eQTLGen datasets. The details of the three comparisons are as below.

Comparison of performance in simulations

To evaluate how well ARCHIE can detect regular *trans*-eQTL signals from a single SNP, we applied ARCHIE to our simulation settings as described in Methods. To recapitulate, we used a real co-expression gene module consisting of 101 genes from DGN dataset. We simulated z-scores from a normal distribution using the correlation matrix of the gene module, sample size 500, causal gene proportion 30% with 30 genes being true target genes, and genetic variance 0.001 as parameters. Trans-PCO has a power of 36% under this setting.

To run ARCHIE, three main inputs are needed[8], including (1) Σ_{GG}, column-correlations of genetic variants, (2) Σ_{EE}, column-correlations of gene expressions, and (3) Σ_{GE}, cross-covariance matrix between variants and gene expressions. In our simulation settings, we tested one variant at a time. Therefore, $\Sigma_{GG} = 1$. We set $\Sigma_{EE} = \Sigma_{101}$, and we approximate Σ_{GE} with $\frac{Z_{101}}{\sqrt{N}}$. We used various genetic variances ranging from 0.002 to 0.006. For each scenario, 1000 simulations were performed.

ARCHIE calculated cc-values (Figure S4B) that measure the *trans* association between each variant and gene sets across 1000 simulations and scenarios. ARCHIE also selected genes (Figure S4C) from the 101 input genes to be target genes in ARCHIE components. While only 30% of genes have true *trans* effects, ARCHIE selected nearly all 101 genes in the ARCHIE component (Figure S4C). To calculate the p-value of a cc-value, we simulated an empirical null distribution of cc-values by simulating one million null z-scores. Then, an empirical p-value is calculated to be the expected number of null cc-values larger than the observed cc-value (Figure S4D). We found that ARCHIE p-values are deflated across genetic variances (Figure

S4D). To calculate power, p-values were adjusted for multiple testing to control the false positive rate using R package ‘qvalue’[9] (FDR < 0.05, Figure S4E).

ARCHIE was not able to identify significant tests in any of 1000 simulations in our simulation settings, even at the largest genetic variance of 0.006 (Figure S4D). This result is not surprising, as ARCHIE is not designed to detect the type of *trans* signal trans-PCO is designed to detect. Trans-PCO is designed to identify weak *trans* effects from a single SNP to a set of genes, for example, from a *cis*-eQTL of a transcription factor gene to multiple co-regulated genes; therefore, in our simulation settings, one SNP has weak *trans* effects on multiple genes with co-regulated expressions. However, ARCHIE is designed to detect *trans* effects where multiple SNPs have weak effects on one gene (for example, multiple GWAS variants have weak *trans* effects on a single gene) or more complicated *trans* effects between multiple SNPs and multiple genes (see Figure 2, Dutta et al.[8]).

Dutta et al.[8] performed simulations to evaluate the power of ARCHIE in simulation settings that ARCHIE is designed for. More specifically, in contrast to our simulation settings, ARCHIE simulations simulated multiple SNPs to have weak *trans* effects on a single gene (the simple model in Figure 2 of Dutta et al.[8]) and a more complex model where multiple SNPs having weak *trans* effects on multiple genes (the complex model in Figure 2 of Dutta et al.[8]). However, the simulation was complicated and was not easy to replicate without the original source code (which is not publicly available). To perform fair comparisons between the two methods, we seeked alternative approaches to evaluate whether trans-PCO could replicate ARCHIE signals in real data analyses (i.e. eQTLGen dataset), rather than in simulations. The details can be found in the following two sections.

Apply trans-PCO to ARCHIE selected gene sets

Another way we used to compare trans-PCO and ARCHIE was to evaluate whether trans-PCO can identify the *trans* signals identified by ARCHIE in the eQTLGen dataset. In eQTLGen, Dutta et al.[8] identified gene sets that are significantly associated with disease–associated variants of 29 traits through *trans* regulation, though only results for three traits were publicly available. Specifically, 2 (resp. 1 and 2) selected gene sets were identified to have significant *trans* associations with prostate cancer (resp. schizophrenia and ulcerative colitis)–associated variants, respectively (Figure S4I). Each component contains a set of variants and a set of genes that are associated through *trans* association. Since trans-PCO is designed to identify *trans*-eQTLs of user-specified gene sets, we applied trans-PCO to gene sets in the five components, and identified the genetic variants associated with the gene sets. We then compared the genetic variants identified by trans-PCO to those by ARCHIE. The goal is to check if trans-PCO could replicate ARCHIE signals.

We applied trans-PCO to eQTLGen summary statistics and used ARCHIE-selected gene sets as gene modules. There are five ARCHIE gene sets for three traits (Figure S4). We performed trans-PCO on the five gene sets and calculated association p-values across all eQTLGen variants. The procedure of applying summary-statistics–based trans-PCO is described in the previous section. To estimate the correlation matrix for a gene set, we used the sample

correlation among genes using independent null variants, which are defined to have p-values smaller than 1e-4 for all genes in the set (Figure S4F). The ratio between the number of null SNPs and number of genes is high (minimum ratio=53), indicating the estimation of the correlation matrix should be accurate enough to avoid false positive inflation (Figure S16 and Figure S17). We test only *trans* variants that are either more than 5Mb away from genes in the set or on different chromosomes, to be consistent to the definition used in Dutta et al.[8]. P-values were adjusted for multiple testing to control the false positive rate by Bonferroni correction (FDR < 0.05, Figure S4G, Figure S4H).

Among five components, four had significant *trans* associations by trans-PCO with at least one of ARCHIE selected variants in the same component (Figure S4H). Therefore, trans-PCO replicated 80% of the *trans* signals identified by ARCHIE at 5% FDR. In total, ARCHIE identified 134 variants in the five components. Trans-PCO replicated a large proportion (min:36.5%~max:85.1%) of the variants for component 1's (C1's) for the three diseases (Figure S4H). Only one out of the 13 variants (7.7%) were replicated by trans-PCO for component 2 (C2) of prostate cancer. Nonetheless, trans-PCO identified 1655 additional significant *trans*-eQTL SNPs at 5% FDR ranging from 65 to 640 for each set (Figure S4G).

In summary, by applying trans-PCO to the five ARCHIE identified gene sets in eQTLGen, we identified 1702 *trans*-eQTL SNPs. ARCHIE identified 134 variants, and 47 (35%) variants are common to both methods. Trans-PCO replicated at least one variant for the corresponding gene set in four out of the five components (Figure S4H). We also found trans-PCO has a better replication of variants in C1's than C2's (36.5%-85.1% in C1 vs. 0%-7.7% in C2).

Comparison of eQTLGen signals

We compared trans-PCO and ARCHIE by directly comparing the eQTLGen signals detected by the two methods. ARCHIE identified gene sets that are significantly associated with disease–associated variants of 29 traits, though only results for three traits were publicly available. There were five significant ARCHIE components for the three traits. Each component contains a set of SNPs and a set of genes, and the set of SNPs are correlated to the set of genes through *trans* regulation.

We also applied trans-PCO to eQTLGen summary statistics as described in the previous section. We analyzed 129 co-expression gene modules and identified 8116 significant *trans*-eSNP–gene co-expression module pairs, corresponding to 2161 eQTLGen test SNPs and 122 gene modules.

To check if ARCHIE signal components found in eQTLGen were replicated by trans-PCO, we checked (1) if ARCHIE selected genes are included in the significant *trans* target gene modules identified by trans-PCO (Figure S4I), and (2) if ARCHIE selected variants are replicated as *trans*-eQTL SNPs by trans-PCO (Figure S4J). Among selected genes by ARCHIE, all of those included in our eQTLGen analysis were included in a significant *trans*-eQTL module by trans-PCO (Figure S4I). Among selected variants by ARCHIE, 31% (40 out of 129 included variants) were also replicated as significant *trans* signals by trans-PCO (Figure S4J). We note

that failing to replicate the remaining ARCHIE signals does not indicate poor performance of trans-PCO for detecting *trans*-eQTLs, as trans-PCO detected 15x more *trans*-eQTL SNPs than ARCHIE. ARCHIE and trans-PCO are designed to detect different *trans* signals: ARCHIE is designed to identify *trans* signals in which a target gene has weak *trans* effects from multiple SNPs (for example from multiple GWAS SNPs to a single gene, as modeled in ARCHIE simulations, Figure 2 of Dutta et al.[8]), whereas trans-PCO is designed to detect *trans* effects from one SNP to multiple genes (for example *trans* effects from a master regulator to multiple downstream genes).

In summary, we compared trans-PCO to ARCHIE in both simulations and in real data analyses. While both are more powerful than the univariate *trans*-eQTL method (Figure 2 of our main text and Figure 2 of Dutta et al.[8]), trans-PCO and ARCHIE are designed to detect different types of weak *trans*-eQTL signals. Our comparison results also support that trans-PCO and ARCHIE are powered at detecting different *trans*-eQTL signals. For example, ARCHIE has no power to detect weak *trans* effects from one SNP to multiple genes in the simulation analyses; while trans-PCO detects a lot more *trans*-eQTL SNPs for the same gene sets than ARCHIE, it only replicate part of the *trans*-eQTL SNPs selected by ARCHIE. There are also other differences between ARCHIE and trans-PCO (see Discussion in the main text), for example, ARCHIE signals are disease specific and the main goal is to identity *trans* genes associated disease associated variants, whereas trans-PCO signals are not disease specific and can be used to perform genome-wide scans of *trans*-eQTLs and produce comprehensive catalogs of *trans*-eQTLs in various tissues and cell types. trans-PCO can be applied to identify *trans*-eQTL SNPs of any user-defined gene sets; in contrast, ARCHIE takes all genes as input and infers a subset of genes *trans* regulated by the variants.

Compare trans-PCO and Rotival et al.

We compared trans-PCO to the method proposed in Rotival et al.[10], which is to identify *trans*-eQTLs of co-expressed gene sets, and shares the same goal as trans-PCO. Therefore, we used simulations to demonstrate the Rotival et al. method has minimal power to identify weak *trans* effects. We will first describe how the Rotival et al. method works and then demonstrate the performance of the method in simulations.

The Rotival et al. method consists of four main steps: (1) Independent Component Analyses (ICA), a matrix factorisation method, is used to infer components representing co-expression patterns from the expression of all genes; (2) ICA components are then tested against all SNPs to filter out non-suggestive associations (p value>1e-7); (3) for the remaining components and SNPs, a subset of genes contributing strongly to each component are selected as a gene module; (4) for a pair of gene module and a SNP, a significant association is identified if the genes in the module are enriched in genes that are individually associated to the SNP (univariate p-value<1e-5) compared to all other background genes outside the module. The enrichment is tested using the hypergeometric test.

We want to note two points in the comparison of Rotival et al. method and trans-PCO. First, filtering out non-suggestive associations in step (2) can lead to loss of power for identifying

trans-eQTLs. Second, enrichment analysis to quantify the associations between a gene module and a SNP has limited power at detecting weak *trans* signals. We elaborate our points as follows.

First, we note that filtering associations of ICA components and SNPs in step 2 can be a major power limiting step. To calculate the association between an ICA component and a SNP, the factor loadings of the ICA component are used as the component (or module) profile. As observed in Kolberg et al.[7], the factor loadings of matrix factorisation (or eigengenes) is highly correlated with the first primary PC (PC1) of gene modules defined by co-expression clustering analysis. However, we and others have shown that PC1 has very limited power for detecting *trans* genetic effects between co-regulated gene sets and variants (see more discussions on PC1 having limited power in the comparison with PC1). Therefore, many *trans* signals would have weak associations with PC1, and thus would be removed from the remaining signals used in following steps to identify final *trans* signals.

Additionally, we note that the enrichment analysis by hypergeometric test is less powerful at detecting weak *trans* effects. As stated above, Rotival et al. essentially uses hypergeometric tests to identify *trans*-eQTL SNP-component associations, which are expected to have an enrichment of weak *trans* effects. We therefore performed simulations to evaluate the performance of the enrichment test used by Rotival et al., assuming genes representing co-expression module are already known. The simulations were adapted from the original simulations evaluating the power of trans-PCO as described in Methods ("Simulation"). We first simulated the z-scores between a SNP and $K = 101$ genes in a gene module, following the distribution $N_K(\sqrt{n}\beta, \Sigma_{K\times K})$, where n is sample size (N = 500), β is a vector representing the true effect sizes of the SNP on K genes and $\Sigma_{K\times K}$is the residualized expression correlation matrix of 101 genes from a real gene module of DGN dataset. Among K genes, a proportion γ of them are causal with non-zero effects. Therefore, we generated β_k from a point normal distribution, where $\beta_k \sim N(0, \sigma^2_b)$ for proportion γ ($\gamma = 1\%, 5\%, 10\%, 30\% \; and \; 50\%$), and $\beta_k = 0$, otherwise. The *trans* genetic variance σ^2_b is set to be 0.001 as default. We also tried larger variances, 0.01, 0.05, 0.1, and 0.2. We simulated 10k SNPs for each simulation and 1k simulations.

To check if target causal genes are enriched in genes included in the module, we also simulated the "background" genes, i.e. genes outside the module. We assume genes outside the module are independent and there are no target causal genes. We simulated 12,001 background genes (12,102 genes used in DGN dataset, subtracted by 101 genes included in the module) from standard normal distribution with zero effects. To define significant individual associations for enrichment, we used p-value cutoff 1e-5 to be consistent with Rotival et al.. Then the enrichment p-value of a SNP for the gene module was calculated by the hypergeometric test. P-values were adjusted using 'qvalue' at FDR<0.1. Power was calculated as the proportion of SNPs that were identified to be significant among 10k SNPs.

As shown in Figure S5, Rotival et al. has minimal power at detecting *trans* associations in the case of weak effects. Under the setting where genetic variance is 0.001, the enrichment test has no power across all causal proportions, while trans-PCO has much higher power, for example, power is 37% when 30% genes are true target genes. We note that the enrichment test can have power for detecting *trans*-eQTLs when the *trans* effects are large (which is not common for *trans*-eQTLs). For example, when genetic variance is 0.01, the enrichment test has a power of 32% when 5% genes are target causal genes. Trans-PCO has a higher power of 64% under the same setting. In the case of even larger genetic variances, e.g. 0.1 and 0.2, the enrichment test has a comparable power with trans-PCO. In summary, the enrichment test does not have power to detect multiple weak *trans* effects.

In summary, we thoroughly compared the performance of trans-PCO versus the three methods. It is clear that trans-PCO significantly outperforms the existing approaches for mapping *trans*-eQTLs of coexpressed gene sets. While the co-regulation patterns of genes by *trans*-eQTLs have long been recognized, trans-PCO provides a powerful and elegant solution to mapping *trans*-eQTLs of these gene sets. Trans-PCO method was carefully made into an easy-to-use and reproducible pipeline. It is flexible and can be applied to both RNA-seq data with genotypes or summary statistics, and the user can define various gene sets (e.g. biological pathways or processes) as modules of interest. Our map of *trans* effects can also be used in follow up analysis, such as colocalization, to improve our understanding how trait associated loci impact gene regulatory networks and pathways through *trans* regulatory effects.

Method S3. Summary statistics based trans-PCO (Related to STAR Methods)

In the case where only summary statistics are available, in order to perform multivariate association test between a SNP and a gene module, we approximated Σ of the module by calculating the sample covariance matrix of z-scores over a large set of independent null SNPs[4] (see previous section). More specifically, we selected independent SNPs that are insignificantly associated (P < 1e-4, Figure S20) with all genes in the module, collected the z-scores of genes over these independent null SNPs, and calculated the sample correlation matrix.

We applied trans-PCO to eQTLGen summary statistics[5]. Specifically, we grouped eQTLGen genes into 166 co-expression modules as defined using DGN dataset. There are only 10,317 trait associated SNPs analyzed in eQTLGen that have full summary statistics for all genes available. Therefore, we searched for independent null SNPs of modules among the limited set of SNPs. One issue is that less SNPs were found to have insignificant associations with all genes in larger modules, which means a low ratio of independent null SNPs over module size for these modules (Figure S16).

Simulations to evaluate and eliminate signal inflations

We wanted to look into if a low ratio of independent null SNPs over module size can lead to noisy estimation of correlation matrix and inflated signals for larger modules. Therefore, we

performed simulations to evaluate the p-values distribution of null tests given various Σ estimations using a range of ratios.

We chose a gene module of size K from 166 DGN co-expression modules and the corresponding Σ_K estimated by DGN gene expression profiles. We first simulated 10k null SNPs with insignificant associations from $Z_K \sim N(0, \Sigma_K)$ as the test set. We then generated z-score matrix $Z_{m\times K}$ of m null SNPs from Σ_K ($Z_{m\times K} \sim N(0, \Sigma_K)$) as a training set to be used for Σ_K estimation. To look at the how using various ratios of independent null SNPs over module size (m/K) affects signal identification, we estimate a series of $\hat{\Sigma}_K$ using the sample correlation of $Z_{m\times K}$ under various number of null SNPs (m). Lastly, we tested the 10k null SNPs by applying trans-PCO using the estimated $\hat{\Sigma}_K$ by various m/K ratios. To look at how $\hat{\Sigma}_K$ affects trans-PCO p-values, we plotted QQ-plot of p-values of all m/K ratios. We performed simulations for modules of various sizes, including module 1-11, 15, 20, 30, 40, 50, 60, 70, 90, 100, 150, 166. We used various m/K ratios, including 1, 5, 10, 50, 100, 150 (Figure S16).

We observed that low m/K ratio can result in inflated null signals, especially for ratios under 50. Therefore, in order to control signal inflations and avoid false positive signals, we removed those large modules with low m/K ratios under 50 (Figure S16) from the following *trans*-eQTLs detection. As a result, we removed 37 co-expression gene modules and performed trans-PCO on the remaining 129 modules.

Other modifications to summary statistics based trans-PCO

We made several other modifications to trans-PCO to make it feasible when only summary statistics are available. We used a more conservative multiple testing correction method, Bonferroni correction, to correct for multiple testing. It is not possible to use the permutation based correction as in analyzing DGN dataset, because no individual level data is available. Therefore, we corrected p-values by multiplying 10,317 (the number of tested SNPs) and 129 (the number of tested gene modules).

We also removed genes from the module that are located on the same chromosome as the SNP, in order to avoid *cis* effects. Additionally, we removed genes cross-mappable with any *cis* genes within 100kb of the tested SNP. This is to reduce false positive *trans*-eQTLs due to possible sequence errors when calculating summary statistics from RNA-seq reads without carefully filtering out problematic reads.

As a summary, we made a few modifications to summary statistics based trans-PCO, to ensure the *trans* signals are well controlled for test statistics inflation and false positives. First, we estimated the correlation matrix of modules using a large number of independent null SNPs ($P <$ 1e-4). Second, we considered only 129 modules that have accurate estimated correlations ($m/K > 50$) for signal detections. Third, we tested the associations between SNPs and genes in

the module that are on different chromosomes as the SNPs. Fourth, we removed genes from the module that are cross-mappable with any cis genes of the SNP (<100kb).

Method S4. Drug targets are associated with immune-related gene sets in *trans* (Related to STAR Methods)

We focused on the disease allergy, because it is immune-related given our analyzed gene expression datasets are from blood tissue. It has a relatively large number of drug target genes (55 launched targets), 5 of which are near (within 1Mb) allergy associated SNPs in eQTLGen (~10k SNPs used for *trans* analysis). We identified SNPs that are significantly associated with allergy using allergy GWAS summary statistics (Table S10, p-value<5e-8). We then examined whether these 5 drug targets are near any SNPs that have significant *trans* associations with immune-related gene co-expression modules or hallmark gene sets in the eQTLGen dataset. Among a total of 142 gene sets (129 co-expression gene modules and 11 hallmark gene sets) used in eQTLGen analysis, 19 were defined as immune-related. Interestingly, we found that all 5 drug target genes near allergy loci are associated with an immune-related gene set through *trans* regulation. Details of the targets and their associated immune-relevant gene sets can be found in Table S19. While the enrichment of allergy drug targets in *trans*-eQTLs of immune-related gene sets is not statistically significant (P=0.12, Fisher's exact test; Table S20), it is likely due to the small number of drug targets in the analyses. Additionally, it is encouraging to see that the gene sets associated with the drug targets are highly relevant to allergy, for example, B cell receptor signaling pathway is associated with three of the drug targets.

Supplemental Figures

Figure S1. Distribution of eigenvalues of gene module 1. Related to STAR Methods.

Figure S2. Simulation results at various genetic variances, including at extremely low proportions of causal genes. Related to Figure 2.

Figure S3. Quantile-quantile plot of P values from null simulations. Related to Figure 2.

Figure S4. Comparison of trans-PCO and ARCHIE. Related to Figure 2 and Figure 3.

Figure S5. Comparison of trans-PCO and Rotival et al.. Related to Figure 2 and Figure 3.

Figure S6. Trans-PCO analyses of co-expression gene modules in DGN. Related to Figure 3.

Figure S7. Comparison between *trans*-eQTLs detected by trans-PCO and PC1 methods. Related to Figure 3.

Figure S8. Simulation scenario when parameters are large. Related to Figure 2.

Figure S9. Simulation scenario when PC1 has the highest power. Related to Figure 2.

Figure S10. Colocalization of *trans*-eQTLs and *cis*-eQTLs at (A) *NFE2* and (B) *PLAGL1* loci. Related to Figure 3.

Figure S11. Gene ontology enrichment of co-expression gene modules (A) M3 and (B) M4. Related to Figure 3.

Figure S12. 965 *trans*-eSNP-module pairs in DGN associated with 50 MSigDB hallmark gene sets representing well-defined biological processes. Related to Figure 3.

Figure S13. Heritability enrichment of all gene modules in all traits. Related to Figure 4.

Figure S14. Colocalization of *trans*-eQTLs of the heme metabolism and various red blood traits. Related to Figure 4.

Figure S15. Heritability enrichment of gene module M3 in blood traits estimated by S-LDSC. Related to Figure 4.

Figure S16. Summary-statistic–based trans-PCO is well controlled for test statistics inflation. Related to Figure 5.

Figure S17. Ratio of independent null SNPs over module size across 50 MSigDB biological processes. Related to Figure 5.

Figure S18. The trans-PCO P values in eQTLGen are much smaller than in DGN. Related to Figure 5.

Figure S19. Associations at the *ARHGEF3* locus with gene modules in both DGN and eQTLGen. Related to Figure 5.

Figure S20. P value cutoff to define null SNPs for gene modules. Related to STAR Methods.

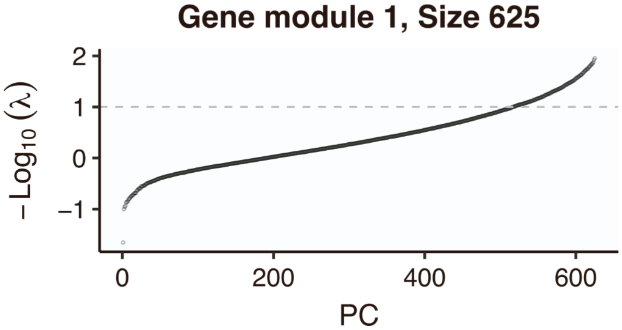

Figure S1. Distribution of eigenvalues of gene module 1. Related to STAR Methods. We use gene modules 1 as an example. PC's are shown on the x-axis. The eigenvalues are shown on the y-axis. The dashed line represents the eigenvalue cutoff (0.01) we used to define PC's included in the test. We can see the last few PC's have extremely small eigenvalues.

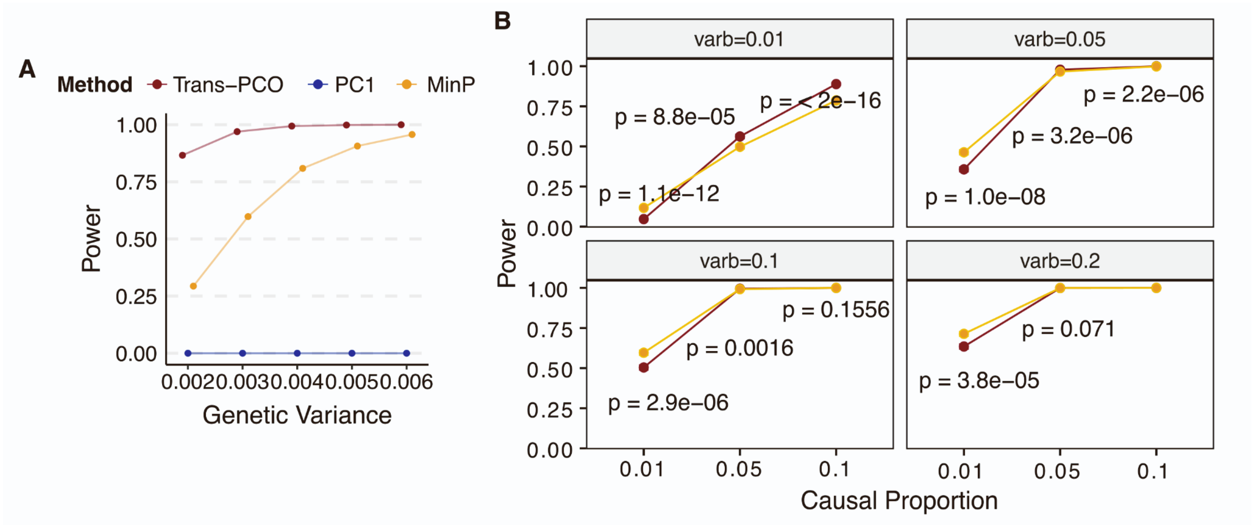

Figure S2. Simulation results at various genetic variances. Related to Figure 2. (A) We compared the power of trans-PCO, PC1, and MinP methods under genetic variances 0.002, 0.003, 0.004, 0.005, 0.006. We used the same gene module as in Figure 2. We simulated sample size to be 500 and the proportion of target genes with non-zero effects in the gene module to be 30%. Power was computed from 10k SNPs across 1000 simulations. The error bars are 95% confidence intervals. Many are too small to be visible. (B) Various genetic variances at extremely low proportions of causal genes. Simulation scenarios when univariate test can be more powerful than multivariate test trans-PCO. We compared the power of trans-PCO and MinP methods under large effect sizes with high levels of sparsity. Specifically, we simulated the proportion of target genes with non-zero effects to be 1%, 5%, and 10%, and large genetic variances to be 0.01, 0.05, 0.1, and 0.2. We used the same gene module as in Figure 2 and simulated the sample size to be 500. Power was computed from 10k SNPs across 1000 simulations. P-values are from the Wilcoxon test to compare two group means. We observe that univariate method ("MinP") has significantly higher power than multivariate method ("trans-PCO") when the sparsity level is high and effect sizes are large. For example, in the case of large genetic variance (varb=0.05) and one gene being causal (casual proportion 1%), MinP has significantly higher power than trans-PCO (p-value=1e-8). As causal genes increase, both MinP and trans-PCO have increased power. To be noted, in the case of small genetic variance (varb=0.01) with weak effects, trans-PCO gains more power than MinP as it aggregates multiple weak effects to improve power.

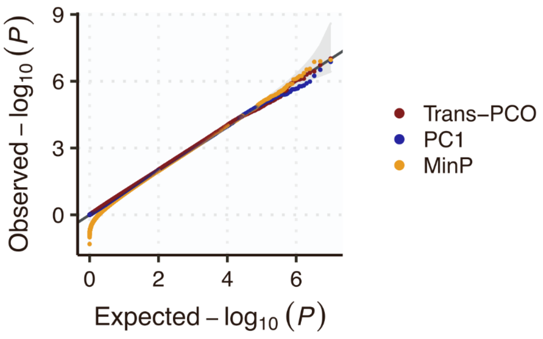

Figure S3. Quantile-quantile plot of P values from null simulations. Related to Figure 2. We simulated 10 million null SNPs with zero effects for genes in the simulated gene module (same as Figure 2 and Figure S2). We tested the *trans* association of simulated SNPs with gene modules using trans-PCO, PC1, and MinP methods and calculated the P values. Colors represent different methods.

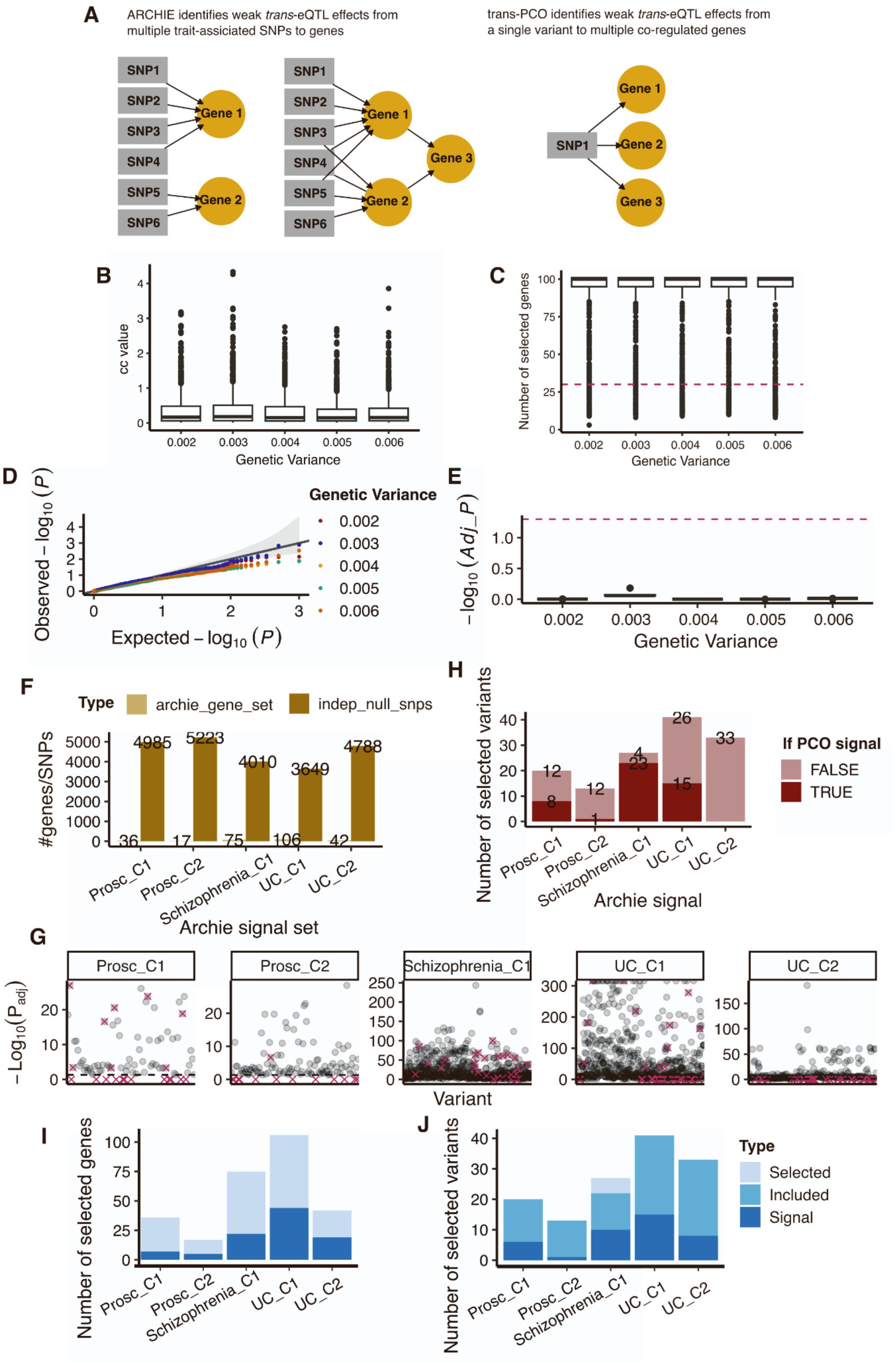
A
ARCHIE identifies weak trans-eQTL effects from multiple trait-assiciated SNPs to genes
trans-PCO identifies weak trans-eQTL effects from a single variant to multiple co-regulated genes
SNP1
SNP2
SNP3
SNP4
SNP5
SNP6
Gene 1
Gene 2
Gene 3
B
cc value
Genetic Variance
C
Number of selected genes
Genetic Variance
D
Observed − log10 (P)
Expected − log10 (P)
Genetic Variance
0.002
0.003
0.004
0.005
0.006
E
− log10 (Adj_P)
Genetic Variance
F
Type
archie_gene_set
indep_null_snps
#genes/SNPs
Archie signal set
Prosc_C1
Prosc_C2
Schizophrenia_C1
UC_C1
UC_C2
H
Number of selected variants
Archie signal
If PCO signal
FALSE
TRUE
G
− Log10(Padj)
Variant
I
Number of selected genes
J
Number of selected variants
Type
Selected
Included
Signal

Figure S4. Comparison of trans-PCO and ARCHIE in Dutta et al.[8]. Related to Figure 2 and Figure 3. (A) Trans-PCO and ARCHIE are designed to capture different *trans* regulatory effects. (B)-(E) Simulation comparison. (B) Distribution of ARCHIE cc-values across various genetic variances. (C) Number of selected genes by ARCHIE across various genetic variances. Red line indicates the number of true target genes, i.e. 30. (D) QQ plot of empirical p-values across various genetic variances. (E) Distribution of adjusted p-values across various genetic variances. Red line shows FDR level 0.05. (E)-(G) Trans-PCO on ARCHIE selected gene sets. (F) X-axis shows significant ARCHIE components for three traits, prostate cancer (Prosc), Schizophrenia, and Ulcerative Colitis (UC). C1 and C2 mean the first and second component. Each component is a pair of selected genes set and variants set. Y-axis shows the size of ARCHIE gene sets (light) and the number of independent null variants (dark) used to estimate correlation matrix of the gene sets. (G) P-values (with Bonferroni correction) of each variant and ARCHIE gene set by trans-PCO. Each panel is an ARCHIE component. Red cross indicates variants selected by ARCHIE. Grey line shows FDR level 0.05. (H) Number of ARCHIE variants across components that are also significant by trans-PCO (dark). (I)-(J) Comparison of eQTLGen signals by trans-PCO and ARCHIE. (I) Number of selected genes across ARCHIE components. Lightest blue (Selected) represents the number of selected genes by ARCHIE of each component. Darker blue (Included) represents the selected genes included in trans-PCO eQTLGen analysis. Darkest (Signal) represents genes included in a significant *trans* target gene module by trans-PCO. All ARCHIE selected genes that are analyzed by trans-PCO are in significant *trans* gene modules by trans-PCO. (J) Number of selected variants across ARCHIE components. Labels are similar as in (I).

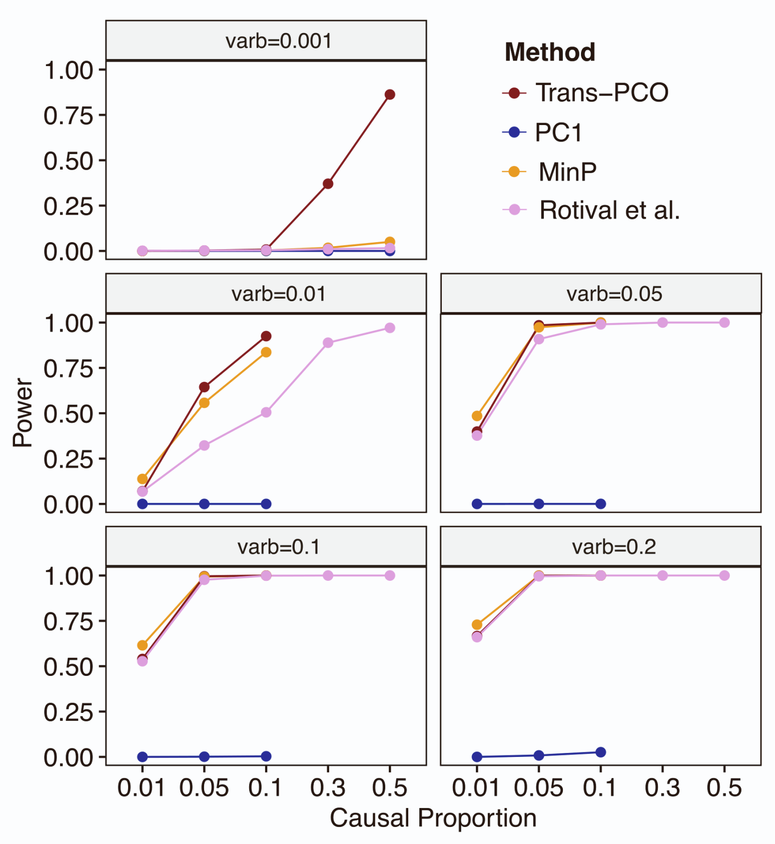

Figure S5. Comparison of trans-PCO and Rotival et al.[10]. Related to Figure 2 and Figure 3. We compared the power of trans-PCO and the method proposed in Rotival et al. across various causal proportions under different genetic variances. Specifically, we simulated the proportion of target genes with non-zero effects to be (1) high levels of sparsity 1%, 5%, 10%, and (2) low levels of sparsity 30%, and 50%, and genetic variances to be (1) small effect 0.001, and (2) large effect 0.01, 0.05, 0.1, and 0.2. We used the same gene module as in Figure 2 and simulated the sample size to be 500. Rotival et al. method used a hypergeometric test to calculate enrichment p-values. P-values were corrected by 'qvalue' to control false positive rate at 10%. Power was computed from 10k SNPs across 1000 simulations.

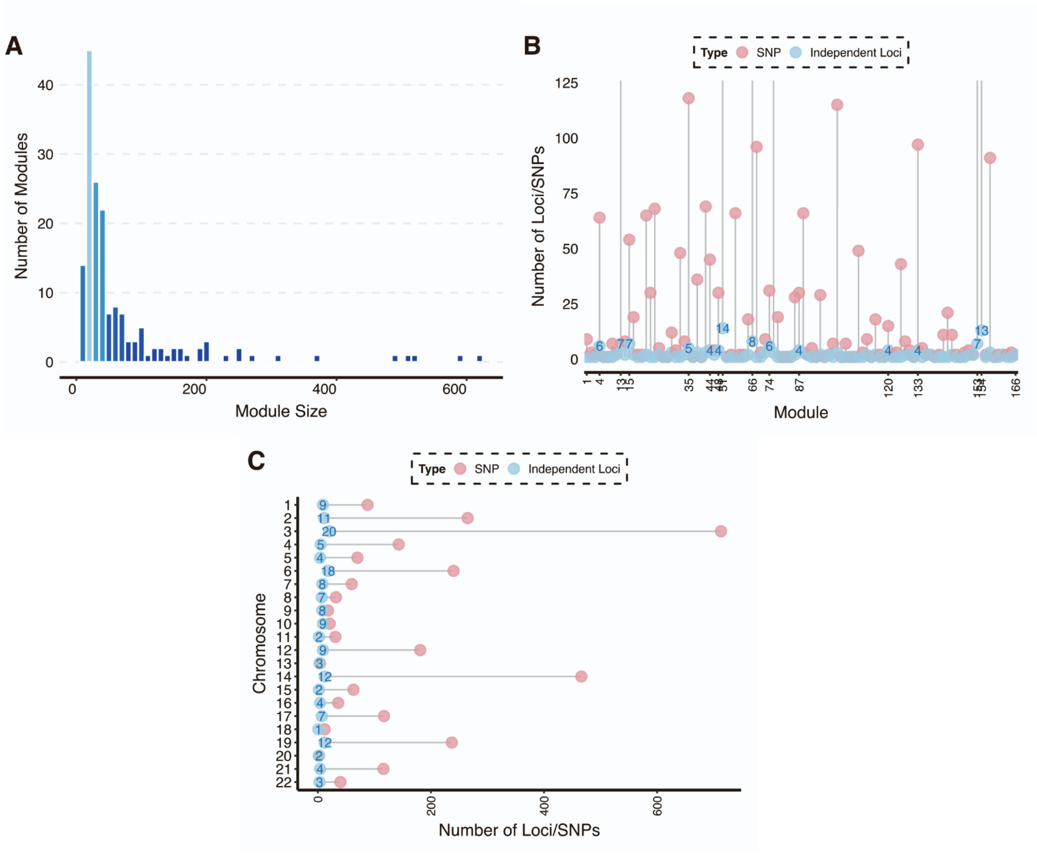

Figure S6. Trans-PCO analyses of co-expression gene modules in DGN. Related to Figure 3. (A) Size distribution of co-expression gene modules. (B)-(C) The number of *trans*-eQTL signals associated with co-expression modules (B) per module and (C) per chromosome. Red points represent the number of *trans*-eSNPs. Blue points represent the number of LD independent loci (R2<0.2).

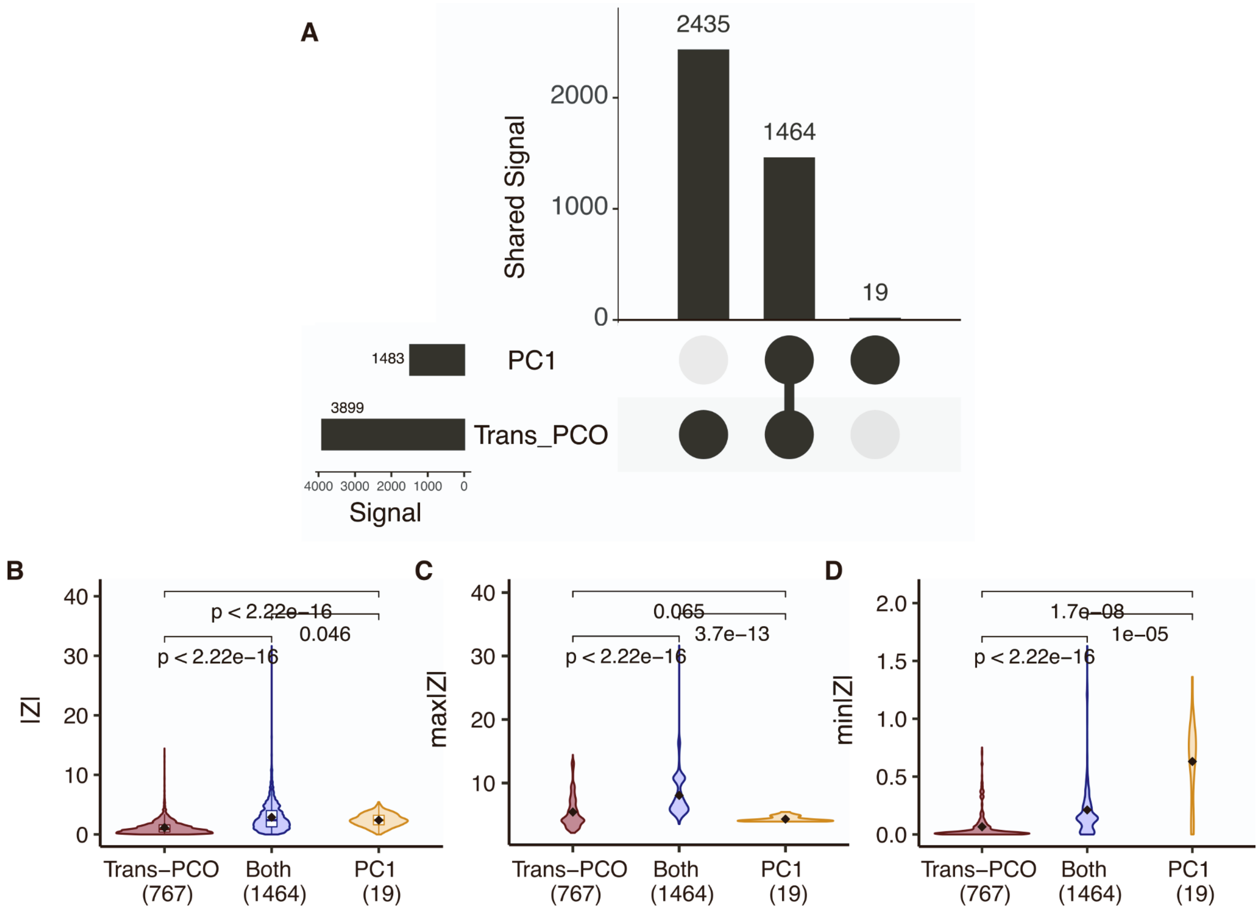

Figure S7. Comparison between *trans*-eQTLs detected by trans-PCO and PC1 method. Related to Figure 3. (A) Signal comparison of PC1 and trans-PCO. The first column shows there are 2435 *trans*-eSNP-module signal pairs that are detected only by trans-PCO not by PC1. The second column shows there are 1464 *trans* pairs identified by both trans-PCO and PC1. The last column shows 19 *trans* signals are identified only by PC1. The horizontal bars on the left show the total number of *trans* signals identified by trans-PCO and PC1, respectively. (B)-(D) Z-scores comparison of PC1 and trans-PCO signals. Mean comparisons were performed by Wilcoxon test. We divided the *trans*-QTLs into three categories, trans-PCO specific signals in red, trans-PCO and PC1 shared signals in blue, and PC1 specific signals in yellow. We compared the z-scores of the three types of *trans*-eQTLs, in terms of (B) the absolute z-scores of signals for all genes in gene modules, (C) the maximum absolute z-scores of signals across genes in gene modules, (D) the minimum absolute z-scores of signals across genes in gene modules. We can see PC1 specific signals have higher z-scores than signals detected by only trans-PCO or both methods, supporting that trans-PCO can detect much weaker *trans* genetic effects.

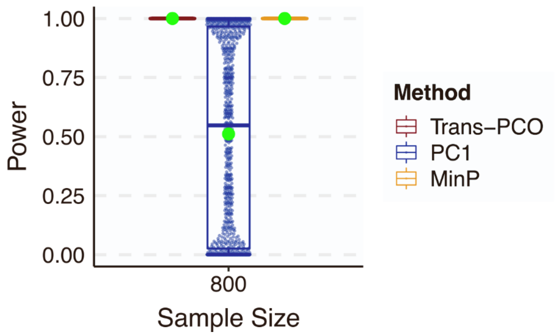

Figure S8. Simulation scenario when parameters are large. Related to Figure 2. We used the same gene module as in Figure 2. We simulated the genetic variance to be as large as 0.2, the proportion of target genes with non-zero effects in the gene module to be 100%, and the sample size to 800. Power was computed from 10k SNPs across 1000 simulations. Green points represent the mean power. Each dot represents a simulation.

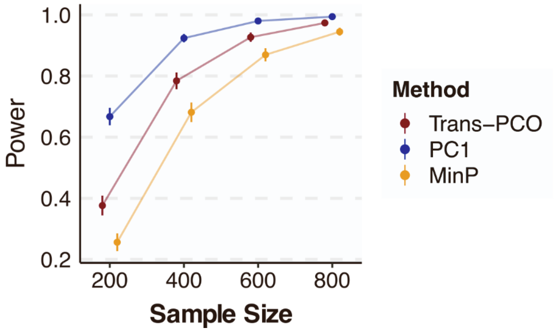

Figure S9. Simulation scenario when PC1 has the highest power. Related to Figure 2. As proved in Liu et al.[4], PC1 method performs best in the case where the effects vector of SNP on genes in the module align with the first eigenvector of the gene module. We used the same gene module as in Figure 2. We simulated the effect of SNPs on genes in the module to be $\sqrt{\sigma_b^2}\mu_1$, where σ_b^2 is the genetic variance with value 0.001, μ_1 is the first eigenvector of the gene module. We simulated the sample size to be 500, and the proportion of target genes with non-zero effects in the gene module to be 30%. Power was computed from 10k SNPs across 1000 simulations. The error bars are 95% confidence intervals.

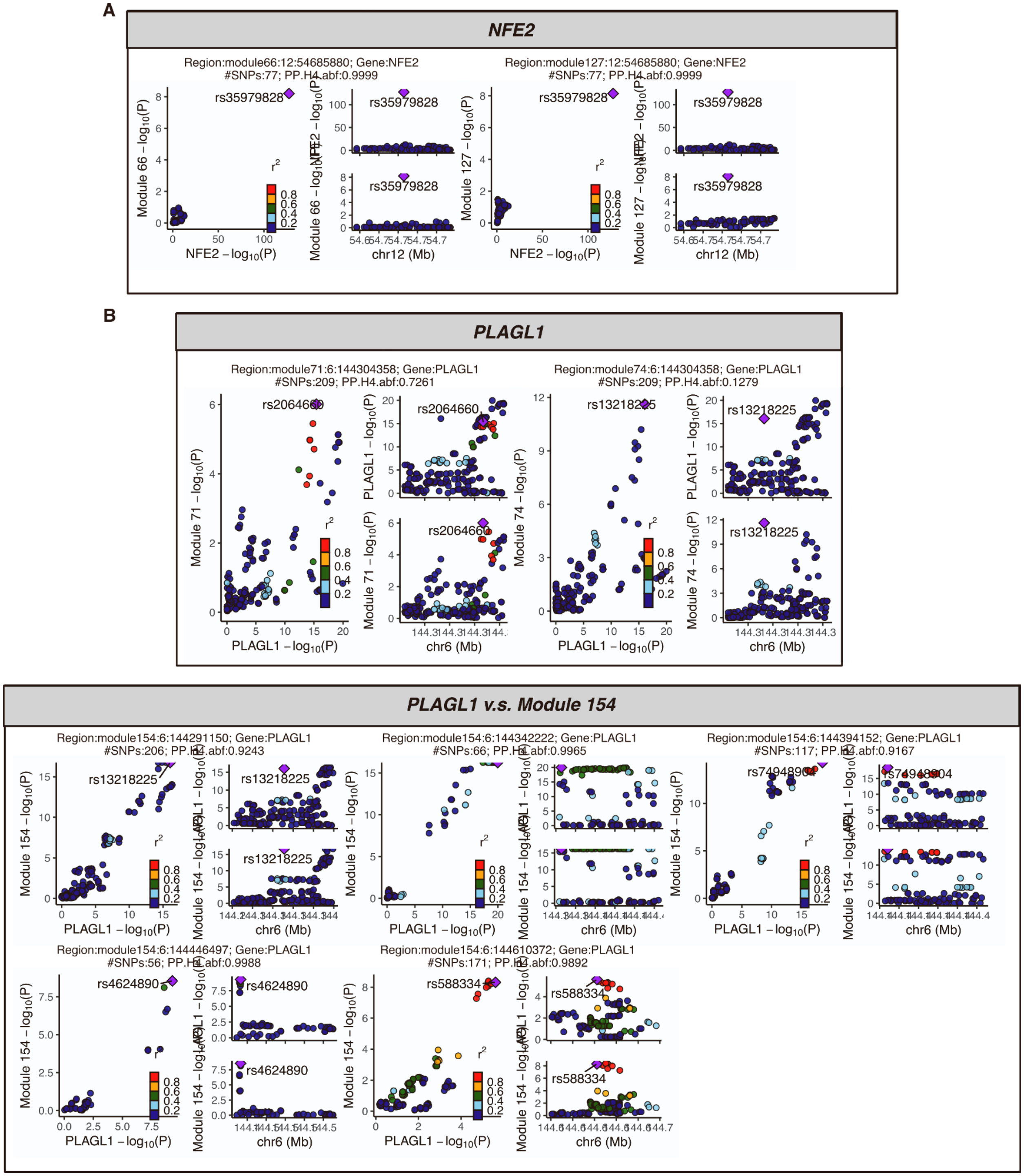
A
NFE2
Region:module66:12:54685880; Gene:NFE2
#SNPs:77; PP.H4.abf:0.9999
Region:module127:12:54685880; Gene:NFE2
#SNPs:77; PP.H4.abf:0.9999
rs35979828
NFE2 – log10(P)
chr12 (Mb)
Module 66 – log10(P)
Module 127 – log10(P)
B
PLAGL1
Region:module71:6:144304358; Gene:PLAGL1
#SNPs:209; PP.H4.abf:0.7261
Region:module74:6:144304358; Gene:PLAGL1
#SNPs:209; PP.H4.abf:0.1279
rs2064660
rs13218225
PLAGL1 – log10(P)
chr6 (Mb)
Module 71 – log10(P)
Module 74 – log10(P)
PLAGL1 v.s. Module 154
Region:module154:6:144291150; Gene:PLAGL1
#SNPs:206; PP.H4.abf:0.9243
Region:module154:6:144342222; Gene:PLAGL1
#SNPs:66; PP.H4.abf:0.9965
Region:module154:6:144394152; Gene:PLAGL1
#SNPs:117; PP.H4.abf:0.9167
Region:module154:6:144446497; Gene:PLAGL1
#SNPs:56; PP.H4.abf:0.9988
Region:module154:6:144610372; Gene:PLAGL1
#SNPs:171; PP.H4.abf:0.9892
rs13218225
rs74948904
rs4624890
rs588334
Module 154 – log10(P)
r2
0.8
0.6
0.4
0.2

Figure S10. Colocalization of *trans*-eQTLs and *cis*-eQTLs at (A) *NFE2* and (B) *PLAGL1* loci. Related to Figure 3. Each sub plot represents a genomic region that has a shared causal variant for the corresponding *trans* target gene module and *cis* gene. The plot title gives (1) the coloc region, which is defined as the *trans* target gene module and the lead *trans*-eQTL in this region, (2) the *cis* gene near the *trans*-eQTL, (3) the number of SNPs in the region, (4) PP4.

A

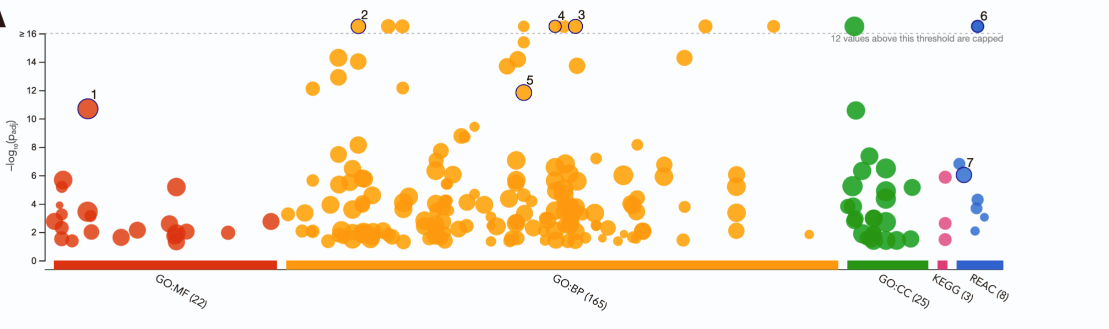

ID	Source	Term ID	Term Name	p_{adj} (query_1)
1	GO:MF	GO:0005515	protein binding	2.039×10^{-11}
2	GO:BP	GO:0009615	response to virus	3.176×10^{-27}
3	GO:BP	GO:0051607	defense response to virus	1.413×10^{-30}
4	GO:BP	GO:0048525	negative regulation of viral process	9.596×10^{-18}
5	GO:BP	GO:0045087	innate immune response	1.469×10^{-12}
6	REAC	REAC:R-HSA-9...	Interferon alpha/beta signaling	4.571×10^{-18}
7	REAC	REAC:R-HSA-1...	Cytokine Signaling in Immune system	9.350×10^{-7}

B

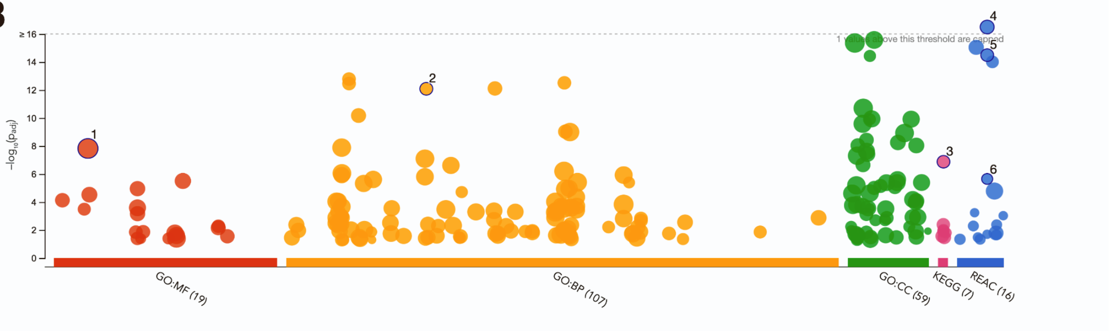

ID	Source	Term ID	Term Name	p_{adj} (query_1)
1	GO:MF	GO:0005515	protein binding	1.495×10^{-8}
2	GO:BP	GO:0030168	platelet activation	8.163×10^{-13}
3	KEGG	KEGG:04611	Platelet activation	1.328×10^{-7}
4	REAC	REAC:R-HSA-7...	Platelet activation, signaling and aggregation	1.062×10^{-21}
5	REAC	REAC:R-HSA-1...	Platelet degranulation	3.229×10^{-15}
6	REAC	REAC:R-HSA-7...	Platelet Aggregation (Plug Formation)	2.241×10^{-6}

Figure S11. Gene ontology enrichment of co-expression gene modules (A) M3 and (B) M4. Related to Figure 3. We used four term categories to look at the enrichment in gene modules[6], including GO:MF, GO:BP, KEGG, and REAC. Categories are shown in colors. The y-axis shows the adjusted enrichment p-values. We highlighted a few most significant and interesting enrichment terms.

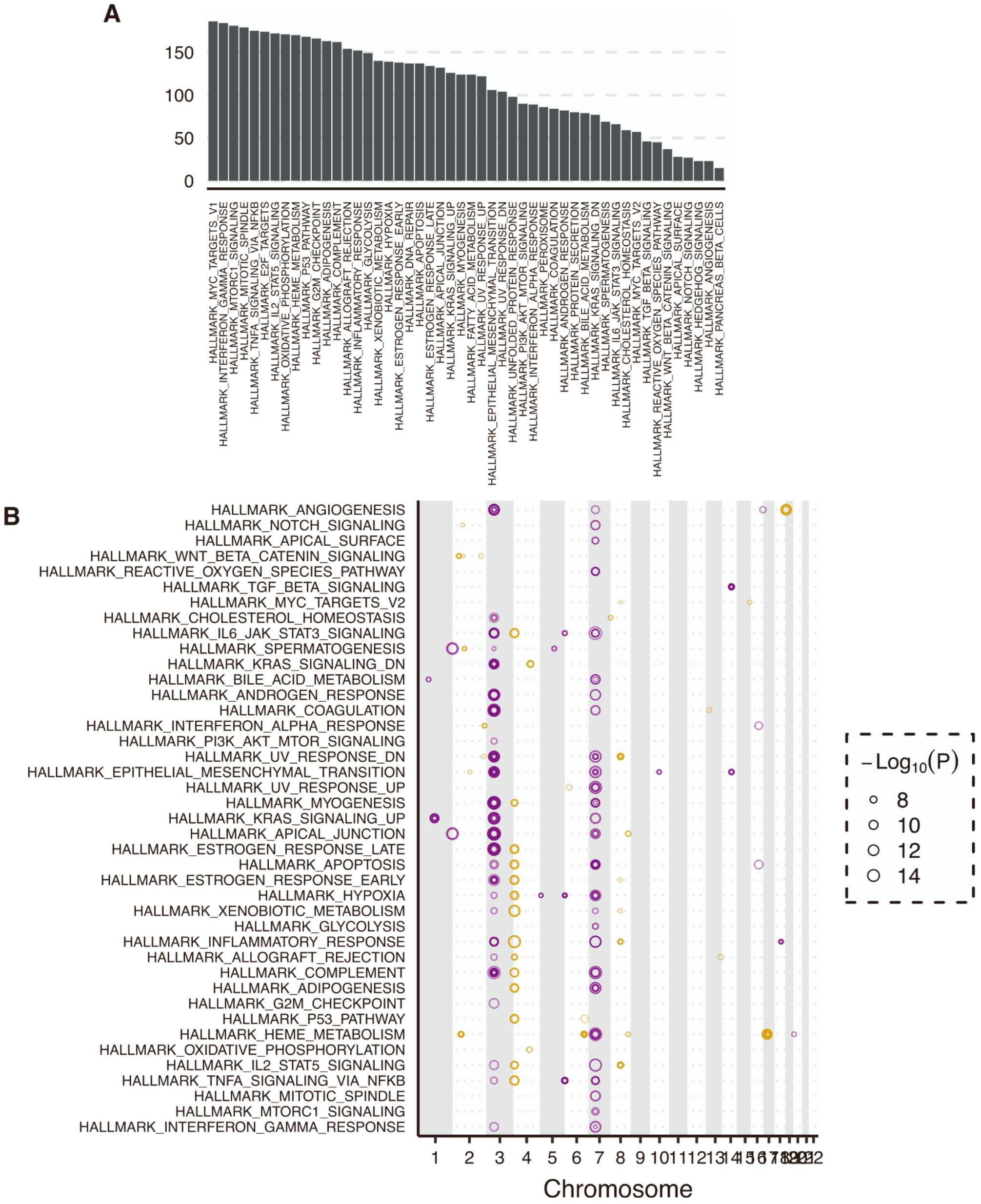

Figure S12. 965 *trans*-eSNP-module in DGN associated with 50 MSigDB hallmark gene sets representing well-defined biological processes. Related to Figure 3. (A) Number of genes in the hallmark gene sets. Names of the biological processes are shown on the x-axis. Sizes are shown on the y-axis. (B) *Trans*-eQTLs of the biological processes. The gene sets are shown on the y-axis. The chromosome position of their corresponding *trans*-eQTLs is on the x-axis. Colors represent odd and even chromosomes to better distinguish the positions. Point size represents the P value of each pair of (*trans*-eSNP, gene set).

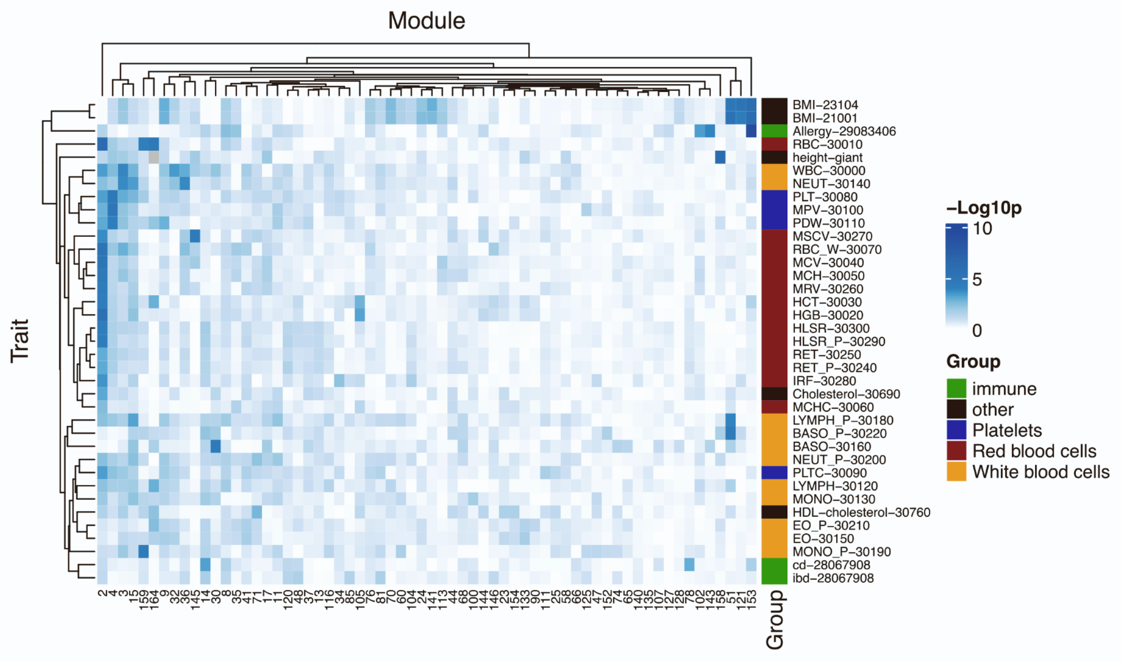

Figure S13. Heritability enrichment of all gene modules in all traits. Related to Figure 4. Each row represents a trait. Traits are colored based on the trait types, including white blood cell traits, red blood cell traits, platelet traits, immune diseases, and other traits. Gene modules are shown on the x-axis. We only show the modules that have at least one colocalized region with a trait. Each tile shows the P values of trait heritability enrichments. The rows and columns are clustered based on the heritability enrichment. We can see traits of the same type share similar heritability enrichment patterns.

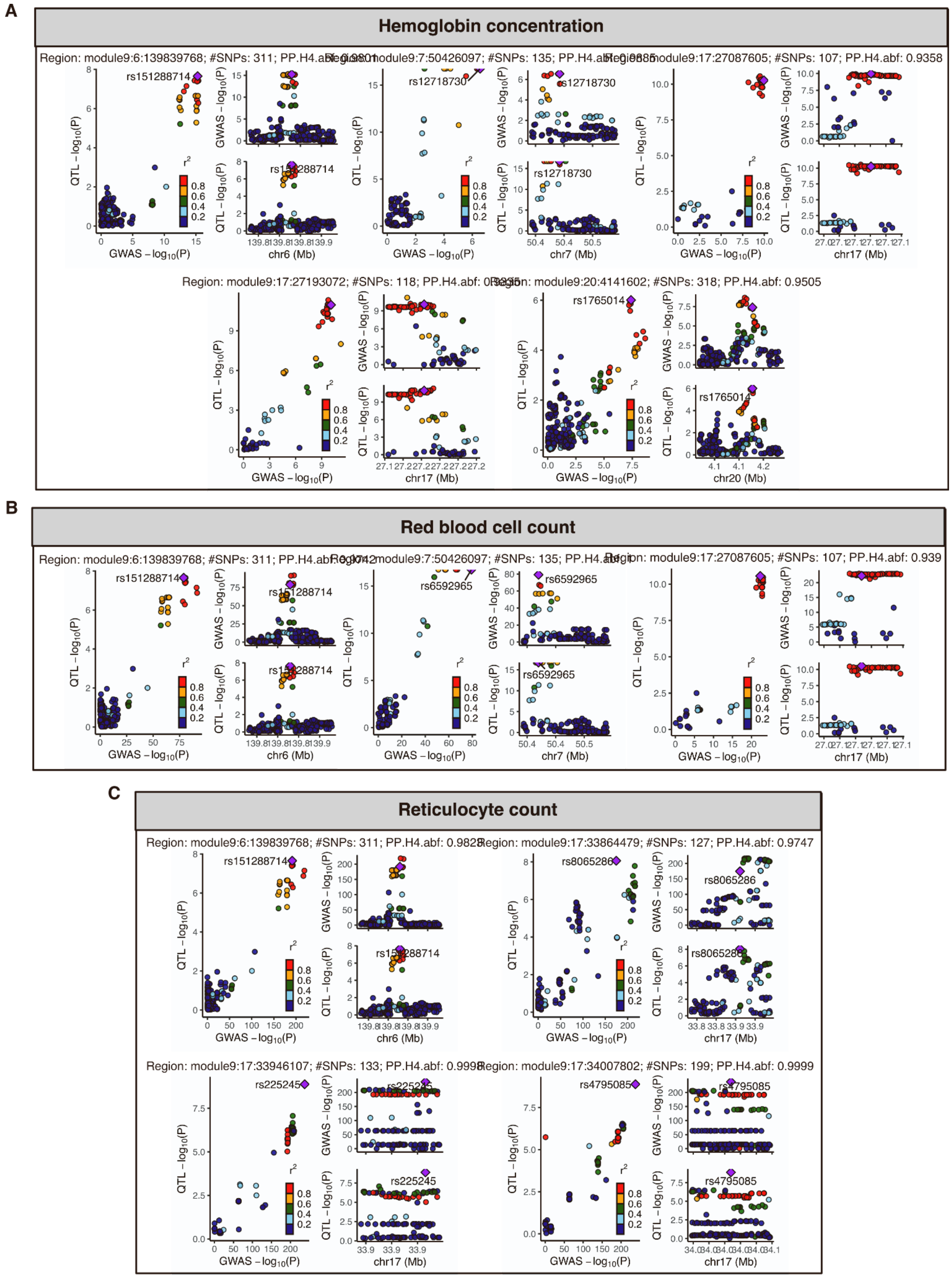
A
Hemoglobin concentration
Region: module9:6:139839768; #SNPs: 311; PP.H4.abf: 0.9801
Region: module9:7:50426097; #SNPs: 135; PP.H4.abf: 0.9835
Region: module9:17:27087605; #SNPs: 107; PP.H4.abf: 0.9358
Region: module9:17:27193072; #SNPs: 118; PP.H4.abf: 0.9365
Region: module9:20:4141602; #SNPs: 318; PP.H4.abf: 0.9505
B
Red blood cell count
Region: module9:6:139839768; #SNPs: 311; PP.H4.abf: 0.9742
Region: module9:7:50426097; #SNPs: 135; PP.H4.abf
Region: module9:17:27087605; #SNPs: 107; PP.H4.abf: 0.939
C
Reticulocyte count
Region: module9:6:139839768; #SNPs: 311; PP.H4.abf: 0.9828
Region: module9:17:33864479; #SNPs: 127; PP.H4.abf: 0.9747
Region: module9:17:33946107; #SNPs: 133; PP.H4.abf: 0.9998
Region: module9:17:34007802; #SNPs: 199; PP.H4.abf: 0.9999
rs151288714
rs12718730
rs1765014
rs6592965
rs8065286
rs225245
rs4795085
QTL −log10(P)
GWAS −log10(P)

Figure S14. Colocalization of *trans*-eQTLs of the heme metabolism and various red blood traits. Related to Figure 4: (A) hemoglobin concentration, (B) red blood cell count, (C) reticulocyte count. The sub plots show colocalization of *trans*-eQTLs of heme metabolism and GWAS loci. The plot title gives (1) the coloc region, which is defined as the *trans* target gene module and the lead *trans*-eQTL in this region, (2) the number of SNPs in the region, (3) PP4.

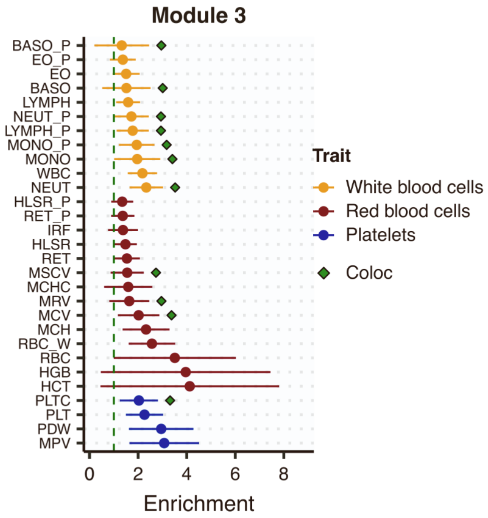

Figure S15. Heritability enrichment of gene module M3 in blood traits estimated by S-LDSC. Related to Figure 4. The y-axis shows the blood traits. Colors represent trait types. The heritability enrichment in module 3 is shown on the x-axis. Error bars represent 95% confidence intervals. Green points indicate that there is significant colocalization of the gene module 3 and the trait.

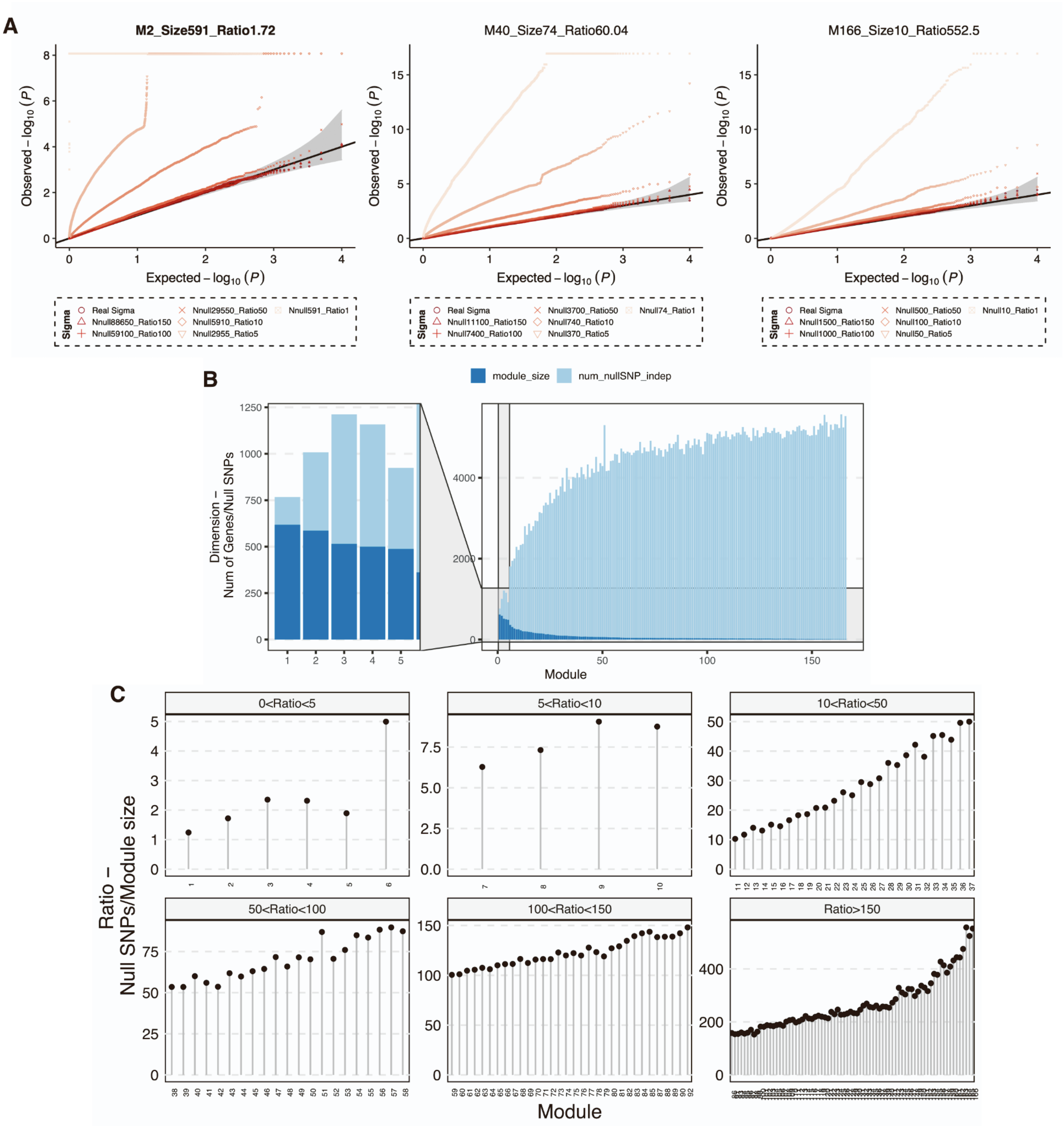
A
M2_Size591_Ratio1.72
M40_Size74_Ratio60.04
M166_Size10_Ratio552.5
Observed – log10 (P)
Expected – log10 (P)
Sigma
Real Sigma
Nnull88650_Ratio150
Nnull59100_Ratio100
Nnull29550_Ratio50
Nnull5910_Ratio10
Nnull2955_Ratio5
Nnull591_Ratio1
Nnull11100_Ratio150
Nnull7400_Ratio100
Nnull3700_Ratio50
Nnull740_Ratio10
Nnull370_Ratio5
Nnull74_Ratio1
Nnull1500_Ratio150
Nnull1000_Ratio100
Nnull500_Ratio50
Nnull100_Ratio10
Nnull50_Ratio5
Nnull10_Ratio1
B
module_size
num_nullSNP_indep
Dimension – Num of Genes/Null SNPs
Module
C
0<Ratio<5
5<Ratio<10
10<Ratio<50
50<Ratio<100
100<Ratio<150
Ratio>150
Ratio – Null SNPs/Module size
Module

Figure S16 Summary-statistic–based trans-PCO is well controlled for test statistics inflation. Related to Figure 5. (A) Noisy correlation matrices lead to trans-PCO test statistics inflation. In addition to the gene module 1 in Figure 5A, we also looked at other gene modules with different sizes to investigate how the noisy correlation matrix estimation affects the signal inflation. Here, we show a few more gene modules, including module 2 with size 591, module 40 with size 74, and module 166 with size 10. The correlation estimation by different ratios of null SNPs over the module size is represented by different point shapes and shades. We observe inflated null P values when correlation matrices were less accurately estimated by lower ratios (of null SNPs over module size). (B) Size of co-expression gene modules and the number of null SNPs used to estimate the correlation matrix. Light blue bar shows the module size. Dark blue bar shows the number of independent null SNPs found in eQTLGen that were used to estimate the correlation matrix of the gene module. (C) Ratio of null SNPs over module size across all co-expression gene modules.

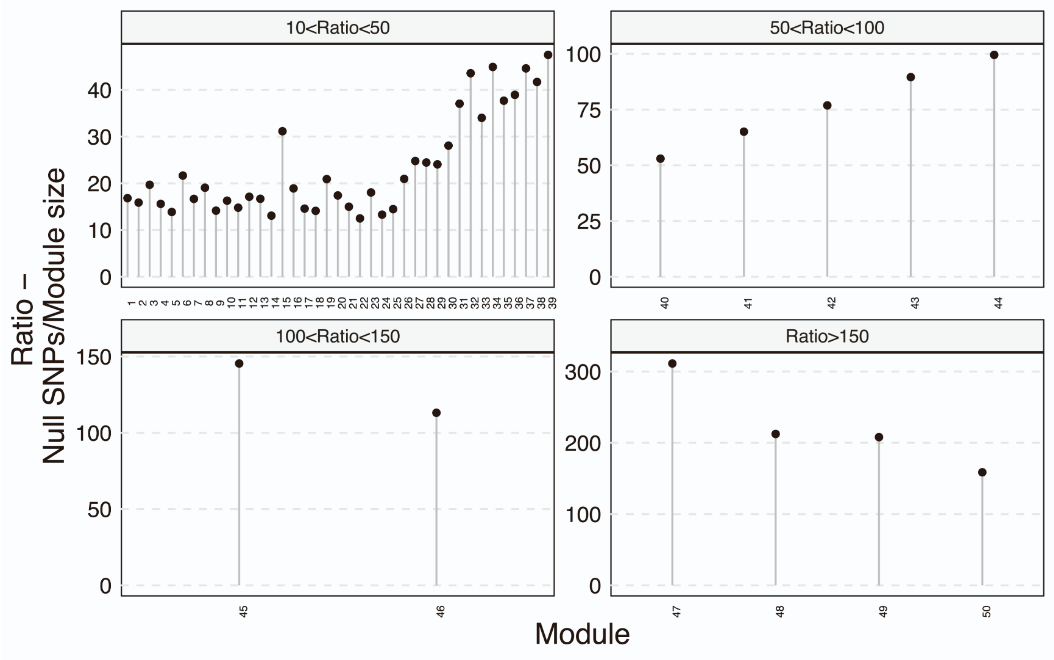

Figure S17. Ratio of independent null SNPs over module size across 50 MSigDB biological processes. Related to Figure 5. The gene sets are shown on the x-axis. The ratio is shown on the y-axis. The gene sets are divided into four categories representing different ratios.

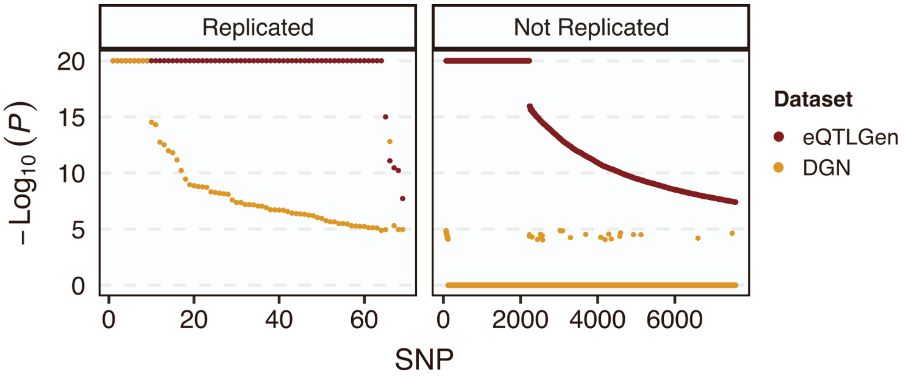

Figure S18. The trans-PCO P values in eQTLGen are much smaller than in DGN. Related to Figure 5. We compared the P value of the same pair of SNP and gene module in DGN and eQTLGen. SNP-module pairs are shown on the x-axis. P values are on the y-axis. DGN and eQTLGen P values are colored in yellow and red, respectively. We divided the SNPs into two categories, (1) SNPs identified as *trans*-eQTLs in both DGN and eQTLGen (left panel, "Replicated"), (2) SNPs identified as *trans*-eQTLs only in eQTLGen not in DGN (right panel, "Not Replicated"). We can observe that, first, trans-PCO P values in eQTLGen are smaller than P values in DGN. Second, the replicated *trans*-QTLs have much smaller P values in DGN than those not replicated.

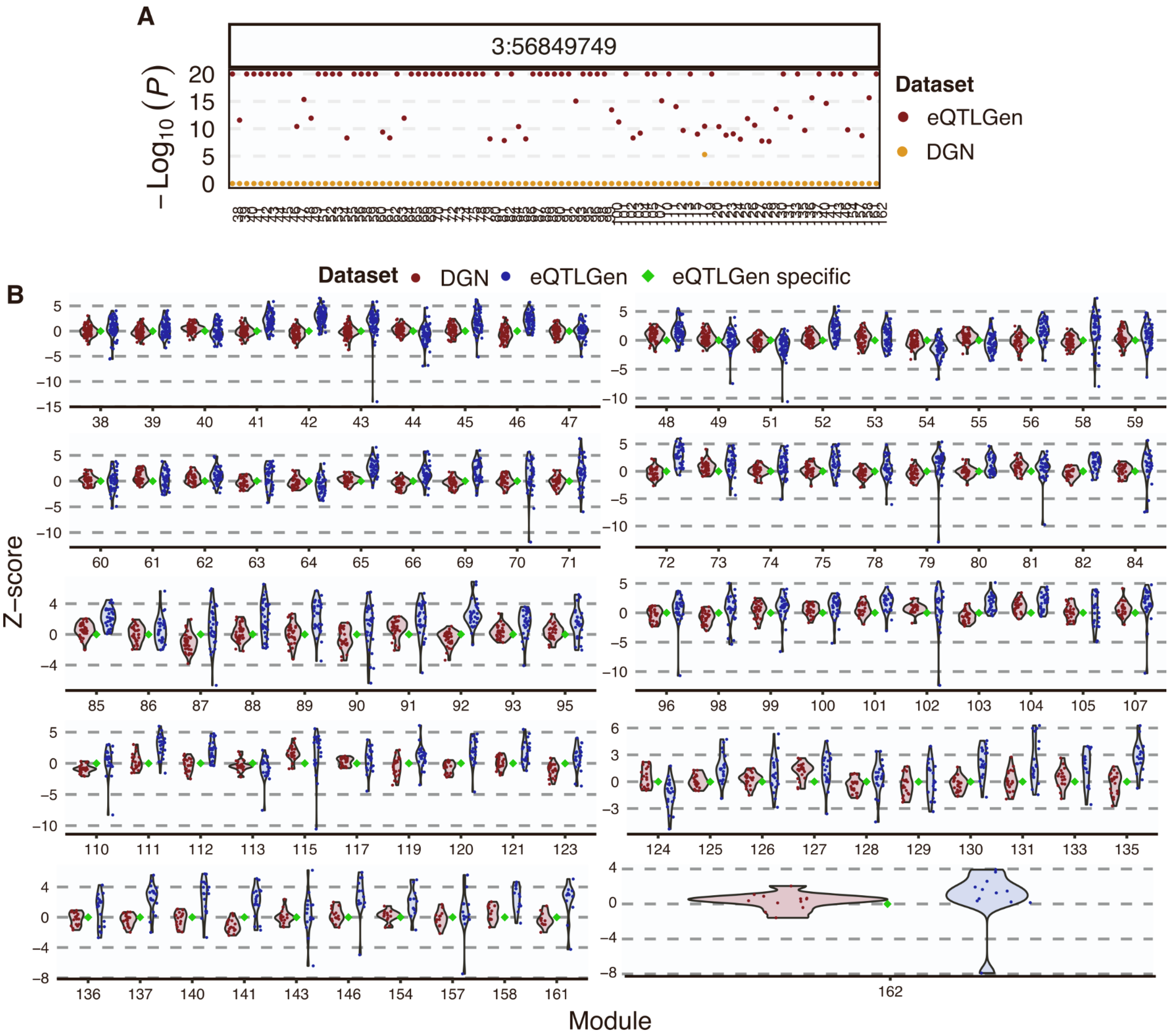

Figure S19. Associations at the *ARHGEF3* locus with gene modules in both DGN and eQTLGen. Related to Figure 5. (A) Associations P values of SNP 3:56849749 at the *ARHGEF3* locus with gene modules analyzed in eQTLGen. The Y-axis shows the association P values in eQTLGen (red) and DGN (yellow). Gene modules are on the x-axis. This SNP has much smaller P values in eQTLGen than in DGN. (B) Z-scores in both DGN and eQTLGen of SNP 3:56849749 across genes in gene modules. The X-axis shows gene modules. The Y-axis shows z-scores of SNP 3:56849749 with the genes in the corresponding module. DGN and eQTLGen z-scores are shown in red and blue, respectively. Green dot indicates that the SNP and the gene module is a signal specific to eQTLGen.

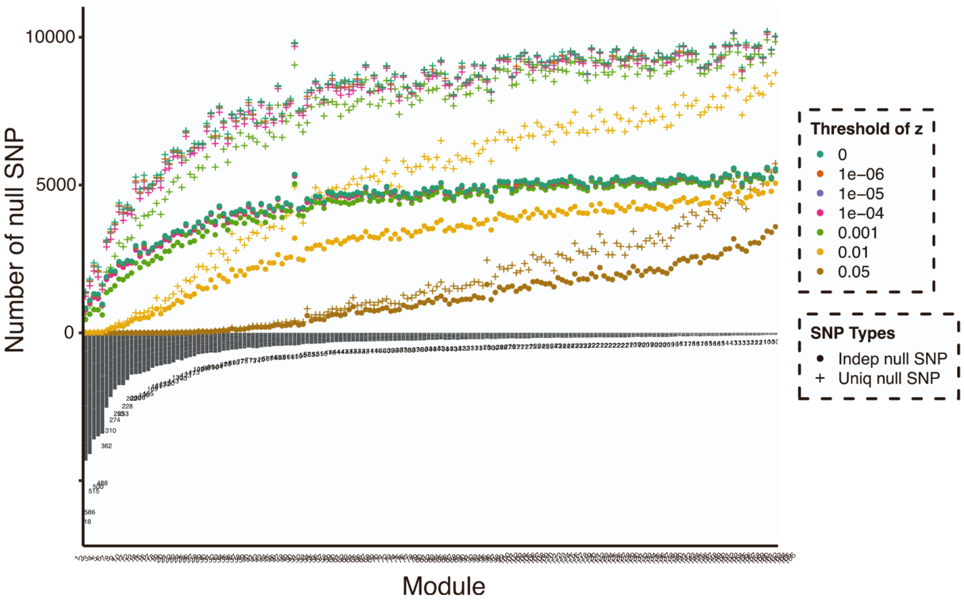

Figure S20. P value cutoff to define null SNPs for gene modules. Related to STAR Methods. We show the gene modules on the x-axis. The module sizes are shown on the lower y-axis. The upper y-axis represents the number of null SNPs in eQTLGen that are insignificantly associated with all genes in a gene module. Cross shapes represent the null SNPs. Circles represent the LD independent null SNPs (R2<0.2). Colors show different p-value cutoffs, 0, 1e-6, 1e-5, 1e-4, 1e-3, 1e-2, and 0.05, to define null SNPs.

Supplemental Tables

Table S1. 166 co-expression gene modules in DGN. Related to Figure 3.

Table S2. Simulation results in Figure 2. Related to Figure 2.

Table S3. A summary of the number of reported signals. Related to Figure 3 and Figure 5.

Table S4. 3899 significant *trans*-eSNP-modules pairs in DGN at 10% FDR. Related to Figure 3.

Table S5. Colocalization of *trans*-eQTLs with *cis*-eQTLs and *cis*-sQTLs in DGN. Related to Figure 3.

Table S6. Gene ontology enrichment of the nearest genes of *trans*-eQTL loci in DGN. Related to Figure 3.

Table S7. Functional annotations of 166 gene co-expression modules. Related to Figure 3.

Table S8. 50 MSigDB hallmark gene sets representing well-defined biological states and processes. Related to Figure 3.

Table S9. 965 significant *trans*-eSNP-modules pairs in DGN dataset with 50 MSigDB gene sets. Related to Figure 3.

Table S10. The list of complex traits and diseases used in colocalization analyses with *trans*-eQTLs. Related to Figure 4.

Table S11. The number and proportion of *trans*-eQTLs that colocalized with each complex trait and disease in Figure 4A. Related to Figure 4.

Table S12. The colocalization results of 179 *trans* loci with complex traits. Related to Figure 4.

Table S13. Heritability enrichment of blood traits in gene module 4 in Figure 5C. Related to Figure 4.

Table S14. The colocalization results of *trans*-eQTL loci of 50 MSigDB hallmark gene sets with complex traits. Related to Figure 4.

Table S15. 8116 (*trans*-eSNP, gene module) pairs detected by trans-PCO in eQTLGen. Related to Figure 5.

Table S16. 2051 significant *trans*-eSNP-modules pairs for 11 MSigDB gene sets in eQTLGen. Related to Figure 5.

Table S17. 38 *trans*-eQTL signals in DGN that are replicated in eQTLGen. Related to Figure 5.

Table S18. Gene ontology enrichment of the nearest genes of *trans*-eQTLs loci in eQTLGen. Related to Figure 5.

Table S19. Allergy drug target genes and their *trans* associated immune-related gene sets. Related to STAR Methods.

Table S20. Enrichment of allergy drug targets in *trans* loci associated with immune-relevant gene sets. Related to STAR Methods.

Table S2. Simulation results in Figure 2. Related to Figure 2.

scenario	paras	method	power_mean	lower_ci	upper_ci
Sample Size	200	Trans-PCO	0.0098943	0.008427764	0.011360836
Sample Size	200	PC1	1.33E-05	1.08E-05	1.58E-05
Sample Size	200	MinP	1.12E-04	7.79E-05	0.000145123
Sample Size	400	Trans-PCO	0.2147116	0.203212419	0.226210781
Sample Size	400	PC1	1.61E-05	1.33E-05	1.89E-05
Sample Size	400	MinP	0.0062486	0.005042673	0.007454527
Sample Size	600	Trans-PCO	0.5188894	0.503889077	0.533889723
Sample Size	600	PC1	1.38E-05	1.12E-05	1.64E-05
Sample Size	600	MinP	0.0491296	0.044182503	0.054076697
Sample Size	800	Trans-PCO	0.7361665	0.723252058	0.749080942
Sample Size	800	PC1	1.76E-05	1.47E-05	2.05E-05
Sample Size	800	MinP	0.1478824	0.138155834	0.157608966
Causal Proportion	0.01	Trans-PCO	1.87E-05	1.57E-05	2.17E-05
Causal Proportion	0.01	PC1	1.30E-05	1.06E-05	1.54E-05
Causal Proportion	0.01	MinP	1.26E-04	2.78E-05	0.000224956
Causal Proportion	0.05	Trans-PCO	0.0021243	0.001133117	0.003115483
Causal Proportion	0.05	PC1	1.41E-05	1.15E-05	1.67E-05
Causal Proportion	0.05	MinP	0.0014167	0.000806123	0.002027277
Causal Proportion	0.1	Trans-PCO	0.0072896	0.005513034	0.009066166
Causal Proportion	0.1	PC1	1.23E-05	9.85E-06	1.48E-05
Causal Proportion	0.1	MinP	0.0029539	0.002246816	0.003660984
Causal Proportion	0.3	Trans-PCO	0.35979	0.345857683	0.373722317
Causal Proportion	0.3	PC1	1.43E-05	1.17E-05	1.69E-05
Causal Proportion	0.3	MinP	0.0184236	0.01608758	0.02075962
Causal Proportion	0.5	Trans-PCO	0.8571084	0.847769449	0.866447351
Causal Proportion	0.5	PC1	1.44E-05	1.16E-05	1.72E-05
Causal Proportion	0.5	MinP	0.0539102	0.04969944	0.05812096
Genetic Variance	0.002	Trans-PCO	0.8662095	0.856884557	0.875534443
Genetic Variance	0.002	PC1	1.57E-05	1.27E-05	1.87E-05
Genetic Variance	0.002	MinP	0.2936808	0.280171348	0.307190252
Genetic Variance	0.003	Trans-PCO	0.9694021	0.965355387	0.973448813
Genetic Variance	0.003	PC1	1.47E-05	1.18E-05	1.76E-05
Genetic Variance	0.003	MinP	0.5978182	0.583051657	0.612584743
Genetic Variance	0.004	Trans-PCO	0.9937744	0.992491074	0.995057726
Genetic Variance	0.004	PC1	1.85E-05	1.51E-05	2.19E-05
Genetic Variance	0.004	MinP	0.8088198	0.79711119	0.82052841
Genetic Variance	0.005	Trans-PCO	0.9983439	0.997521874	0.999165926
Genetic Variance	0.005	PC1	1.78E-05	1.47E-05	2.09E-05
Genetic Variance	0.005	MinP	0.9064	0.898358151	0.914441849
Genetic Variance	0.006	Trans-PCO	0.9997041	0.999550519	0.999857681
Genetic Variance	0.006	PC1	1.93E-05	1.58E-05	2.28E-05
Genetic Variance	0.006	MinP	0.9570192	0.952202712	0.961835688

Table S3. A summary of the number of reported signals. Related to Figure 3 and Figure 5.

	**Note**	**DGN Dataset**		**eQTLGen Dataset**	
		**Co-expression gene modules**	**MSigDB hallmark gene sets**	**Co-expression gene modules**	**MSigDB hallmark gene sets**
(trans-eSNP, module)	Number of pairs of a SNP with significant trans association and its corresponding target gene module	3899	965	8116	2051
(LD independent trans-loci, module)	Number of pairs of a gene module and the LD independent loci of its corresponding trans-eSNPs	202	120	NA	NA
trans-eSNP	Number of SNPs with significant trans associations across all gene modules	2955	411	2161	1018
LD independent trans-loci	Number of LD independent loci of trans-eSNPs with significant trans associations across all gene modules	145	43	NA	NA

Table S19. Allergy drug target genes and their *trans* associated immune-related gene sets. Related to STAR Methods.

Drug target gene	Gene set type	Gene set index	Selected gene set annotation (see full annotations in Table S7 and Table S8)
SLC37A4	Co-expression module	M54	B cell receptor signaling pathway
UGT3A1	Co-expression module	M54;M62;M76;M87	B cell receptor signaling pathway;TNFR1-induced NFkappaB signaling pathway;NF-kappa B signaling pathway;regulation of T cell activation
IL3	Co-expression module	M54;M108	B cell receptor signaling pathway;Antigen processing and presentation
IL3	Hallmark gene set	HALLMARK_IL6_JAK_STAT3_SIGNALING;HALLMARK_NOTCH_SIGNALING	Genes up-regulated by IL6 via STAT3, e.g., during acute phase response;Genes up-regulated by activation of Notch signaling
ATP5B	Hallmark gene set	HALLMARK_IL6_JAK_STAT3_SIGNALING	Genes up-regulated by IL6 via STAT3, e.g., during acute phase response
FGF1	Hallmark gene set	HALLMARK_IL6_JAK_STAT3_SIGNALING	Genes up-regulated by IL6 via STAT3, e.g., during acute phase response

Table S20. Enrichment of allergy drug targets in *trans* loci associated with immune-relevant gene sets. Related to STAR Methods.

Fisher's exact test P: 0.12		
	Allergy drug targets near allergy loci in eQTLGen	Non-allergy drug targets near loci in eQTLGen
Genes near trans-eQTL of immune-related gene sets	5	6054
Genes near trans-eQTL of non-immune-related gene sets	0	3180

Supplemental References

1. Battle, A., Mostafavi, S., Zhu, X., Potash, J.B., Weissman, M.M., McCormick, C., Haudenschild, C.D., Beckman, K.B., Shi, J., Mei, R., et al. (2014). Characterizing the genetic basis of transcriptome diversity through RNA-sequencing of 922 individuals. Genome Res. *24*, 14–24. 10.1101/gr.155192.113.
2. Taylor-Weiner, A., Aguet, F., Haradhvala, N.J., Gosai, S., Anand, S., Kim, J., Ardlie, K., Van Allen, E.M., and Getz, G. (2019). Scaling computational genomics to millions of individuals with GPUs. Genome Biol. *20*, 228. 10.1186/s13059-019-1836-7.
3. Liu, X., Mefford, J.A., Dahl, A., He, Y., Subramaniam, M., Battle, A., Price, A.L., and Zaitlen, N. (2020). GBAT: a gene-based association test for robust detection of trans-gene regulation. Genome Biol. *21*, 211. 10.1186/s13059-020-02120-1.
4. Liu, Z., and Lin, X. (2019). A Geometric Perspective on the Power of Principal Component Association Tests in Multiple Phenotype Studies. J. Am. Stat. Assoc. *114*, 975–990. 10.1080/01621459.2018.1513363.
5. Võsa, U., Claringbould, A., Westra, H.-J., Bonder, M.J., Deelen, P., Zeng, B., Kirsten, H., Saha, A., Kreuzhuber, R., Yazar, S., et al. (2021). Large-scale cis- and trans-eQTL analyses identify thousands of genetic loci and polygenic scores that regulate blood gene expression. Nat. Genet. *53*, 1300–1310. 10.1038/s41588-021-00913-z.
6. Raudvere, U., Kolberg, L., Kuzmin, I., Arak, T., Adler, P., Peterson, H., and Vilo, J. (2019). g:Profiler: a web server for functional enrichment analysis and conversions of gene lists (2019 update). Nucleic Acids Res. *47*, W191–W198. 10.1093/nar/gkz369.
7. Kolberg, L., Kerimov, N., Peterson, H., and Alasoo, K. (2020). Co-expression analysis reveals interpretable gene modules controlled by trans-acting genetic variants. eLife *9*. 10.7554/eLife.58705.
8. Dutta, D., He, Y., Saha, A., Arvanitis, M., Battle, A., and Chatterjee, N. (2022). Aggregative trans-eQTL analysis detects trait-specific target gene sets in whole blood. Nat. Commun. 13, 4323. 10.1038/s41467-022-31845-9.7
9. Storey JD, Bass AJ, Dabney A, Robinson D (2021). qvalue: Q-value estimation for false discovery rate control. R package version 2.24.0. http://github.com/jdstorey/qvalue.
10. Rotival, M., Zeller, T., Wild, P.S., Maouche, S., Szymczak, S., Schillert, A., Castagné, R., Deiseroth, A., Proust, C., Brocheton, J., et al. (2011). Integrating Genome-Wide Genetic Variations and Monocyte Expression Data Reveals Trans-Regulated Gene Modules in Humans. PLOS Genet. 7, e1002367. 10.1371/journal.pgen.1002367.
